# Skin Fibroblasts from Individuals Self-Diagnosed as Electrosensitive Reveal Two Distinct Subsets with Delayed Nucleoshuttling of the ATM Protein in Common

**DOI:** 10.3390/ijms26104792

**Published:** 2025-05-16

**Authors:** Laurène Sonzogni, Joëlle Al-Choboq, Patrick Combemale, Amélie Massardier-Pilonchéry, Audrey Bouchet, Philippe May, Jean-François Doré, Jean-Claude Debouzy, Michel Bourguignon, Yves Le Dréan, Nicolas Foray

**Affiliations:** 1 Unité Mixte de Recherche (UMR)1296 «Radiation: Defense, Health, Environment», Institut National de la Santé et de la Recherche Médicale (INSERM), Centre Léon-Bérard, 69008 Lyon, France; laurene.sonzogni@inserm.fr (L.S.); joelle.al-choboq@inserm.fr (J.A.-C.); patrick.combemale@lyon.unicancer.fr (P.C.); audrey.bouchet@inserm.fr (A.B.); jean-francois.dore@inserm.fr (J.-F.D.); michel.bourguignon@inserm.fr (M.B.); 2 Department of Dermatology, Centre Léon-Bérard, 69008 Lyon, France; 3 University Claude-Bernard Lyon 1, University Gustave-Eiffel, Unité Mixte de Recherche Epidémiologique et de Surveillance Transport Travail Environnement (UMRESTTE), UMR_T9405, 690008 Lyon, France; amelie.massardier-pilonchery@chu-lyon.fr; 4 Department of Occupational Medicine and Occupational Diseases, Centre Hospitalier Lyon-Sud, Hospices Civils de Lyon, 90008 Lyon, France; 5 Institut de Recherche Biomédicale des Armées, 1 Place du Générale Valérie André, BP 40073, 91223 Brétigny-sur-Orge, France; philippe.may@intradef.gouv.fr (P.M.); jcddebouzy@gmail.com (J.-C.D.); 6 University Paris-Saclay, 78035 Versailles, France; 7 Inserm, Ecole des Hautes Etudes en Santé Publique (EHSEP), Institut de Recherche en Santé, Environnement et Travail (IRSET), University of Rennes, UMR_S 1085, 35000 Rennes, France; yves.le-drean@univ-rennes1.fr

**Keywords:** electrosensitivity, EHS, EMF, radiosensitivity, DNA double-strand breaks, DSB repair, ATM, self-assessment questionnaire, immunofluorescence, oxidative stress

## Abstract

Electromagnetic hyper-sensitivity (EHS) and its causal link with radio-frequencies raise a major question of public health. In the frame of the clinical study DEMETER, 26 adult volunteers self-diagnosed as EHS-positive agreed to reply to a self-assessment questionnaire and to provide a skin biopsy sampling to establish a primary fibroblast cell line. The questionnaire and the biological data revealed, independently, 2 subsets of donors associated each with a low background, highly responsive (LBHR) and a high background, lowly responsive (HBLR) phenotype. A couple of subsets based on questionnaire data and based on the yield of spontaneous DNA double-strand breaks were found to be composed of the same donors at 64% identity. After exposure to X-rays, and application of anti-*γH2AX*, *pATM*, and *MRE11* immunofluorescence, all the DEMETER fibroblasts (26/26) elicited a delayed radiation-induced ATM nucleoshuttling (RIANS). The use of RIANS biomarkers showed that the 2 phenotypes described above corresponded to DEMETER donors with a high risk of cancer (LBHR) or high risk of accelerated aging (HBLR). By exposing DEMETER cells to H_2_O_2_ followed by an antioxidative agent, we confirmed that EHS may be related to the management of DNA strand breaks. A preliminary molecular model of EHS inspired by the RIANS model was proposed.

## 1. Introduction

For several decades, man-made electromagnetic fields (EMF) such as those produced by microwaves whose frequency ranges from 300 MHz to 300 GHz have entered our daily lives [1,2,3,4]. These microwave hyper-frequencies (HF) concern a growing number of applications, present in both professional and general public domains: radio and television broadcasting, mobile telephony, military radars, medical hyperthermia, satellite communications, etc. Still recently, due to considerable technological advances and needs, new HF applications are being developed (Bluetooth, WiFi, WiMax, etc.), which drastically increases the number of HF sources and the global environmental radiation pressure [5,6,7]. In parallel, a strong societal concern has emerged and demanded a better understanding of the potential biological effects of HF and an assessment of the possible associated risks, in order to increase the acceptability of such a technological revolution [8,9,10]. Progressively, some authors have attributed to HF some symptoms like cataracts, fatigue, anxiety, and discomfort revealed by exposed individuals [11,12,13,14,15,16]. To date, the list of potentially HF-induced symptoms is increasing, while the direct proof of the causal link between exposure to HF and the occurrence of these symptoms is still a source of debate [17,18,19,20,21,22,23]. The term “electromagnetic hyper-sensitivity” (EHS) was first proposed to reflect the group of symptoms that some individuals attribute to exposure to EMF. This is notably the case of headache, sleep disturbance, fatigue, difficulty concentrating, dizziness, … [17,24,25]. However, it is noteworthy that some other terms, such as “microwave syndrome”, have been also cited, which did not help exhaustive bibliographical analyses [26]. More recently, a third term, “idiopathic environmental intolerance attributed to EMF” (IEI-EMF), has been proposed to better reflect the diversity of symptoms but also the uncertainty about the nature of their link with EMF [17,27].

Historically, the idea that EMF can impact human health was raised with the discovery of X-rays, at the end of the 19th century. Around 1896, while induction coils (e.g., the Ruhmkorff’s induction coil) were required to increase the voltage for emitting X-rays, the French radiologist Etienne Destot attributed the tissue injuries observed after X-ray exposure to the EMF emitted by the induction coils [28,29]. However, the accumulation of radiopathology data led to the conclusion that some adverse tissue responses can be observed in the absence of coils (i.e., without EMF emission), and therefore were due to ionizing radiation [30]. To date, in literature, there is a consensus to consider that EMF, as non-ionizing radiation, transfers a much lower amount of energy to living matter than ionizing radiation [31]. In other terms, electrosensitivity and radiosensitivity should trigger different biological and clinical features according to the quantity of energy absorbed. It is probably from this paradigm that some authors have called into question the very existence of EMF-induced symptoms and EHS, notably through provocative or double-blind studies [32,33]. EHS has been also compared to “nocebo”, an effect where the belief in a potential negative effect of an exposure causes symptoms, even in the absence of any physical cause, although such a notion is still debated to date [34,35]. Lastly, some studies have also suggested that people suffering from EHS may have a greater propensity to experience symptoms related to stress or anxiety notably through somatization [36]. Some public health organizations, such as the World Health Organization (WHO), recognize EHS as a self-reported phenomenon but emphasize that there is insufficient scientific basis to make it an official medical diagnosis. WHO nevertheless recommends that people experiencing symptoms consult a healthcare professional to assess and treat them [1,2,3,4].

The majority of studies on EHS have focused on individuals and their behavior [17,19]. An alternative approach may be to investigate the EMF response of cells deriving from EHS individuals in order to overcome the biases of behavioral studies. However, such an approach, that, to date, represents a minority of reports, raises two problems, at least: (1) how to select EHS individuals and on which criteria, while the existence of EHS itself with specific symptoms remains controversial? (2) what cellular model should we choose? With regard to the first question, it must be stressed that WHO recommended including, in any clinical study of EHS, a self-assessed evaluation with graded intensities of symptoms or discomfort through a questionnaire [1]. With regard to the second question, our long experience of investigations about individual radiosensitivity led us to favor the use of skin fibroblasts which represent, at least, 5 advantages by comparison to lymphocytes, and other types of cells like astrocytes: (1) fibroblasts are the most representative cells of the human body and connective tissue; (2) skin biopsies established from dermatological punches are easy to perform and the success rate for establishing cell lines is very high; (3) fibroblasts are stable, long-lived in cell culture and do not specifically elicit apoptosis-like lymphocytes; (4) their shape, their contact inhibition property and their propensity to be maintained as monolayers mimic well the tissue scale; (5) some reproducible skin reactions to EMF, like prickling, burning sensations and rashes, have been reported, while it is not the case with blood [37].

**Figure 1:**
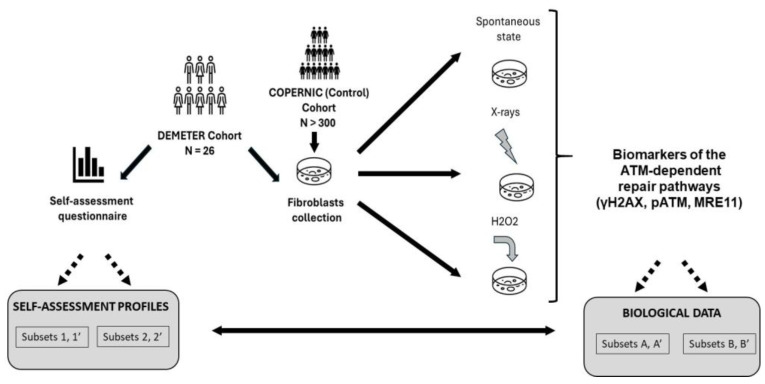
General organization of the DEMETER study. The 26 DEMETER volunteers agreed to fill in a self-assessment questionnaire and to provide a skin biopsy. Spontaneous, X-rays- and H_2_O_2_-induced DSB were assessed in resulting fibroblasts. The COPERNIC data (non-EHS radioresistant or radiosensitive cases) were compared to the DEMETER ones. The potential links between questionnaire and biological data were investigated, notably by comparing the compositions of a couple of subsets of DEMETER donors defined from questionnaire and biological data.

Some symptoms observed in EHS patients like skin rashes may have been also observed in some patients showing cellular and clinical radiosensitivity [38]. Since both cellular and clinical radiosensitivity have been shown to be linked to impaired DNA double-strand breaks (DSB) pathways dependent on the ATM kinase protein, a major actor of the stress response [39,40], it appeared of great interest to investigate the functionality of the ATM-dependent DSB recognition and repair pathways in skin fibroblasts from EHS patients. Hence, in order to provide the first molecular definition of EHS, a medical study, called DEMETER (French acronym for Molecular definition of human electrosensitivity) was conducted with a cohort of 26 self-diagnosed electrosensitive volunteers. The DEMETER study consisted of both a self-assessment questionnaire and molecular investigations on the collection of fibroblast cell lines deriving from the DEMETER donors. These two tasks were performed separately and independently. Another collection of fibroblast cell lines from the lab, called COPERNIC, and its associated radiobiological database provided by cancer patients treated with radiotherapy (RT) who did not describe any EHS symptoms in their questionnaire, were used as non-EHS controls [41]. Among the COPERNIC donors, radioresistant (In this study, the notion of radioresistance is defined both clinically (absence of significant tissue reactions post-RT), at the cellular scale (surviving fraction at 2 Gy, SF2 > 55%) and at the molecular scale (maximal number of pATM foci after 2 Gy, pATMmax > 35 pATM foci). It is noteworthy that the subpopulation of radioresistant individuals represents about 75–85% of individuals [39]) apparently healthy non-EHS donors served as negative controls for EHS, while cells from non-EHS cancer patients treated with RT and showing intermediate radiosensitivity served as negative controls for radiosensitivity [40]. Lastly, the conclusions drawn from the first two steps (questionnaire and biological data) were combined to identify specific molecular features of EHS. In the present report, only the self-assessment questionnaire data and the data obtained after exposure of DEMETER and COPERNIC cells to X-rays and H_2_O_2_, two very documented stress inducers of DNA strand breaks data are presented (Figure 1). EMF-induced data will be the subject of another report. One of the major goals of this study is to define common features of EHS that could be defined from both biological investigations and self-assessment questionnaires. Particularly we focused on the DNA double-strand breaks (DSB) recognition, repair, and signaling in skin fibroblasts from DEMETER patients (Figure 1).

## 2. Results

Two types of investigations were conducted independently:

- a self-assessment questionnaire composed of five parts: questions about the intensities of symptoms and discomfort (A) after exposure to various and current sources of EMF; (B) after exposure to some non-EMF sources; (C) according to the tissue/organ exposed, before exposure to EMF sources; (D) according to the tissue/organ exposed, during exposure to EMF sources, and a free area (P) for personal information to detail eventual medical history and ongoing treatments. For A to D questions, intensities were scored from 0 (the lowest intensity) to 5 (the highest intensity).
- a measurement of the DNA breaks in DEMETER cells at the spontaneous state and in response to X-rays and H_2_O_2_, two documented sources of DNA strand breaks.

In the last step, both analyses were put together to identify some common features and propose, if possible, a first molecular definition of EHS.

### 2.1. Analysis of Data from the Self-Assessment Questionnaire

#### 2.1.1. General Features of the DEMETER Cohort

The DEMETER cohort was composed of 26 self-diagnosed donors as electrosensitive, including 20 (77%) women and 6 (23%) men. The average age of the donors was 52.4 ± 1.6 years at the skin biopsies sampling (minimum: 39; maximum: 69; 52.9 ± 1.4 and 51.8 ± 4.1 years for women and men, respectively).

#### 2.1.2. Sources of EMF That May Induce Symptoms: Definition of the Subsets 1 and 2

**Figure 2:**
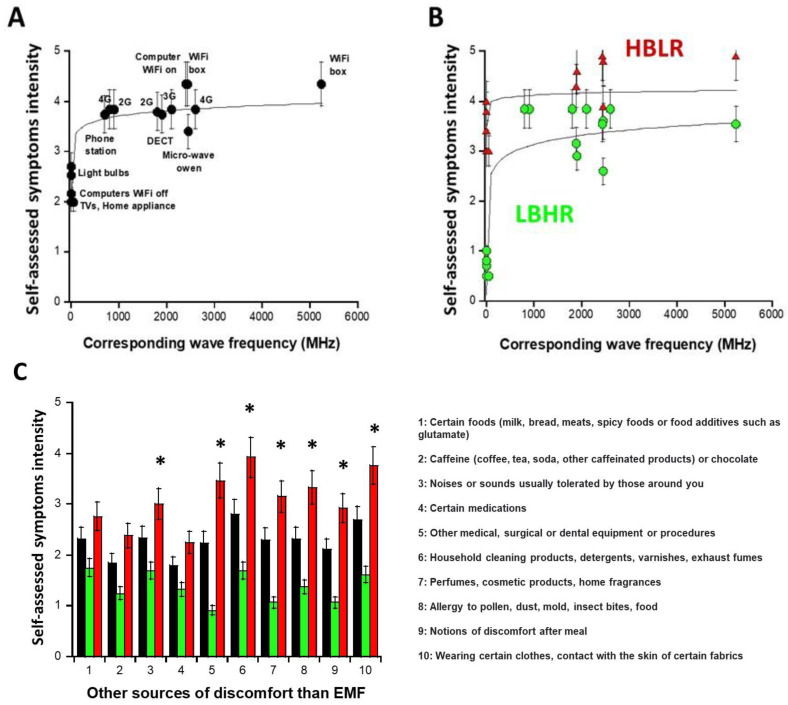
Questionnaire data expressed as self-diagnosed symptoms and discomfort intensities as a function of the wave frequency of EMF sources of interest (**A**,**B**) and of sources different from EMF waves (**C**). Each data is the mean (±standard error of the mean (SEM)) of the values self-assessed by the 26 DEMETER donors (black symbols), by the DEMETER donors of the subset 1 (LBHR) and 2 (HBLR) (green and red symbols, respectively). The logarithmic formulas obtained from data fits were drawn with solid lines ((**A**): Equation (1); (**B**): Equations (2) and (3)). In panel (**C**), the asterisks indicate a significant difference (one-way ANOVA) between subsets 1 and 2 data and mean (*p* < 0.05).

Part A of the self-assessment questionnaire asks DEMETER donors about their possible symptoms and discomfort in response to exposure to various sources of EMF. All the donors of the DEMETER cohort replied to the questions of part A by giving a graded intensity of severity from 0 (no reaction) to 5 (extreme pain and discomfort). For each source of EMF, the average of the self-diagnosed symptom intensities (I_average_) provided by the 26 DEMETER donors was calculated and plotted against the corresponding wave frequencies (WF) expressed in MHz (Figure 2A). The empirical formula providing the best data fit was:

I_average_ (WF) = 2.67 + 0.34 log (WF)  (R = 0.81) (1)

The resulting curve showed that the average intensity of the self-diagnosed symptoms, I_average,_ brutally increased from 300 MHz and reached its maximum in the UHF domain, i.e., in the [300 MHz, 3 GHz] interval with I_average_ (UHF) ≈ 3.92 (Figure 2A). However, for some DEMETER donors, significant self-diagnosed symptoms and discomfort were also described even outside this UHF domain: indeed, by considering only the sources of EMF lower than UHF (i.e., home appliance devices, light bulbs, computers with WiFi off, corresponding to the A5, A6, A7 questions of the self-assessment questionnaire, respectively (Supplementary Figure S1; see Supplementary Data), the average intensity I_A5,A6,A7_ was found to be equal to 2.48. Such a cut-off value permitted to define two distinct subsets of DEMETER donors, namely subsets 1 and 2, obeying the following conditions and formulas, respectively (Figure 2B):

- the subset 1 of DEMETER donors (N = 14; 53.8%) with relatively low self-assessed intensities of symptoms and discomfort when exposed to EMF lower than UHF (I_A5,A6,A7_ < 2.48): numerically, I_A5,A6,A7_ = 0.8 in the [0.7–1] interval. The DEMETER subset 1 donors were also characterized by relatively high self-assessed intensities of symptoms and discomfort when exposed to UHF (2.6 < I_UHF_ < 3.84; I_average_ (UHF) = 3.5; I_average_ (UHF) − I_A5,A6,A7_ = 2.7) (Figure 2B). The subset 1 gathered therefore the DEMETER donors who elicited a low background (LB) but a relatively high self-assessed responsiveness (HR) to exposure to UHF, defining the “LBHR” phenotype. It was the case of the following DEMETER donors: #4, 5, 7, 8, 9, 11, 15, 16, 18, 20, 22, 23, 24, and 25 (Table S1, Supplementary Data), whose data obeyed the following formula:

Subset 1: I_average/subset1_ (WF) = 1.32 + 0.60 log (WF)  (R = 0.45) (2)

- the subset 2 of DEMETER donors (N = 12; 46.1%) with relatively high self-assessed intensities of symptoms and discomfort when exposed to EMF lower than UHF (I_A5,A6,A7_ > 2.48): numerically, I_A5,A6,A7_ = 3.8 in the [3.4–4] interval. The DEMETER subset 2 donors were also characterized by relatively low self-assessed intensities of symptoms and discomfort when exposed to UHF (3.37 < I_UHF_ < 4.71; I_average_ (UHF) = 3.95; I_average_ (UHF) − I_A5,A6,A7_ = 0.15) (Figure 2B). The subset 2 gathered therefore the DEMETER donors who elicited a high background (HB) but a relatively low self-assessed responsiveness (LR) to exposure to UHF defining therefore the “HBLR” phenotype. It was the case of the following DEMETER donors: #1, 2, 3, 6, 10, 12, 13, 14, 17, 19, 21, 26 (Table S1, Supplementary Data), whose data obeyed the following formula:

Subset 2: I_average/subset2_ (WF) = 3.72 + 0.13 log (WF)  (R = 0.85) (3)

It is noteworthy that both subsets 1 and 2 donors were characterized by a similar age at the skin biopsy sampling (51.5 ± 2.0 and 53.5 ± 2.0 years, respectively). The sex ratios were also found similar: 25% and 21.4% men in subsets 1 and 2, respectively (Table S1, Supplementary Data).

#### 2.1.3. Nature of the Non-EMF Sources That May Induce Symptoms

Part B of the self-assessment questionnaire asks DEMETER donors about their possible symptoms and discomfort in response to exposure to various non-EMF sources. All the donors of the DEMETER cohort replied to the questions of part B of the self-assessment questionnaire. As for part A, the average of the self-diagnosed symptom intensities was calculated for each non-EMF source (in black; Figure 2C) but also for the subsets 1 (LBHR) and 2 (HBLR), predefined above (in green and red, respectively, Figure 2C). Among the different non-EMF sources, those triggering the highest intensities of symptoms and discomfort on average were household cleaning products (e.g., detergents) and wearing certain manufactured tissues (Figure 2C). However, the largest I-values were reached for the subset 2 (HBLR) donors and concerned, in addition to the two precited items: medical procedures (#5), allergy (item #8) (suggesting that the subset 2 (HBLR), more than the subset 1 (LBHR) may be associated with a certain immunodeficiency), perfumes (item #4), noises and sounds (#3) and post-meal discomfort (#9) (Figure 2C).

Hence, the data analysis of part B of the questionnaire suggested that the DEMETER subset 1 (LBHR) donors were not particularly sensitive to other sources than EMF while the DEMETER subset 2 (HBLR) donors may be sensitive to other agents than EMF such as contact with household cleaning products or certain manufactured tissues and may show some immunodeficiency.

#### 2.1.4. Medical History and Ongoing Treatments of the DEMETER Donors

Part P of the self-assessment questionnaire asks DEMETER donors about their medical history and ongoing treatments. Only 7 DEMETER donors filled the part P related to their medical history and ongoing treatment (Table S1; Supplementary Data): among them, 5 (71.4%) DEMETER donors belonged to the subset 2 (HBLR), suggesting that, in the present study, the HBLR phenotype is mainly associated with significant medical history and/or ongoing treatment (Table S1; Supplementary Data).

#### 2.1.5. Nature of the Symptoms Before and During the Exposure to EMF: Definition of the Subsets 1′ and 2′

Parts C and D of the self-assessment questionnaire ask DEMETER donors about the intensity of the possible symptoms and discomfort before and during the exposure to EMF sources according to the tissue, respectively. All the donors of the DEMETER cohort replied to the questions of part C. Unfortunately, one donor did not fill in the form concerning part D (intensity of symptoms and discomfort during the exposure to EMF). Since the reason for such omission is unknown, the C and D data from this DEMETER donor (#24) were excluded from the calculations.

**Figure 3:**
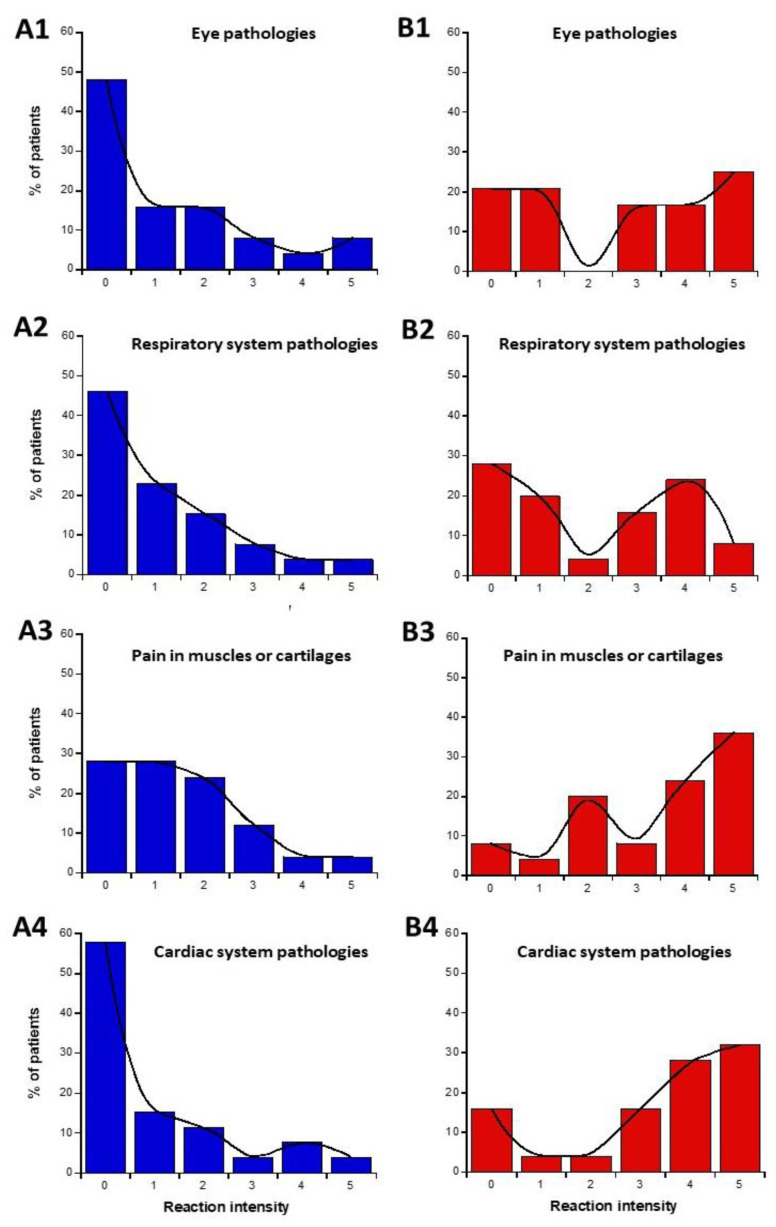

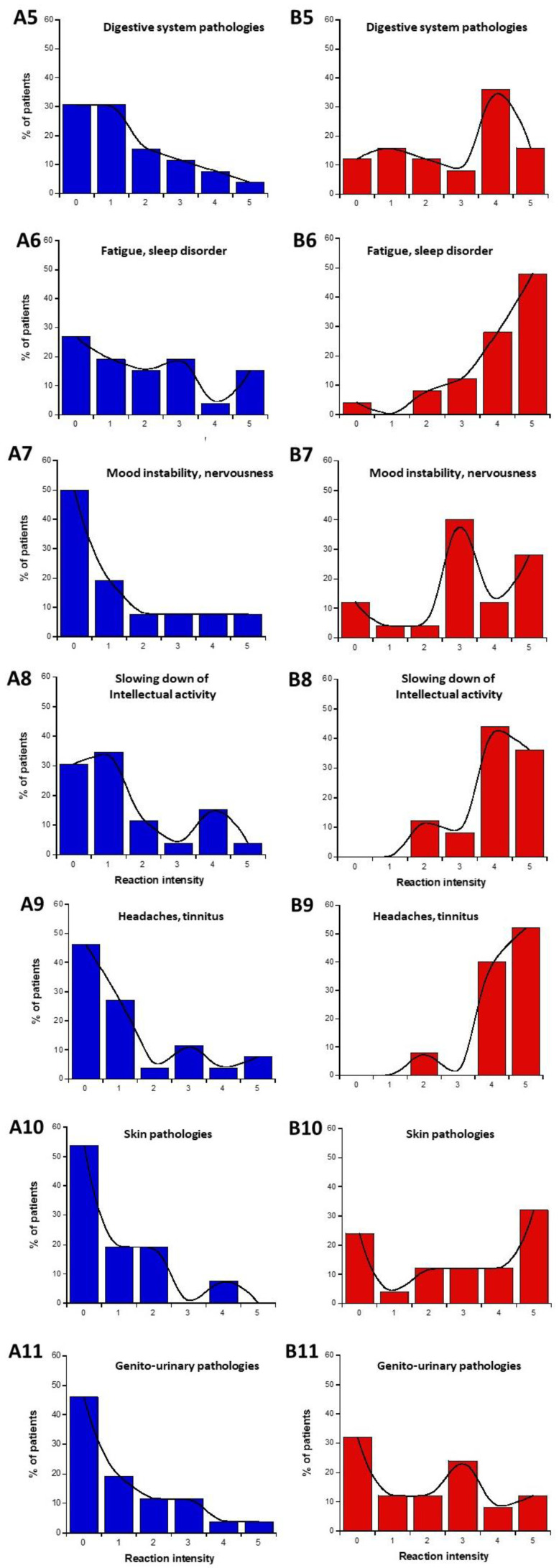
Data of the questionnaire parts C and D, expressed as distribution I-histograms of the self-assessed intensities of symptoms or discomfort before (**A**) or in response to EMF sources (**B**). for the indicated organs or pathologies. Solid line represents the smooth fit of the distribution data.

The data from parts C and D of the self-assessment questionnaire were plotted as intensities I-distribution histograms for all the organs and pathologies indicated: eye pathologies, pain in muscles and cartilages, cardiac system pathologies, digestive system pathologies, fatigue and sleep disorders, mood instability, and nervousness, slowing down of intellectual activity, headaches, and tinnitus, skin pathologies, genito-urinary pathologies (Figure 3). The replies to the C and D parts of the questionnaire were represented in blue and red (Figure 3A and Figure 3B, respectively).

We asked whether a definition of two specific subsets of DEMETER donors was possible from parts C and D of the self-assessment questionnaire. We called these two subsets 1′ and 2′. Considering the diversity of responses and conditions, the data analysis of the I-histograms shown in Figure 3 was performed by obeying the following steps:

- Calculation of the average of the self-assessed I-intensities of symptoms and discomfort before (part C) or in response to the exposure to EMF sources (part D) for each organ/pathology.
- Estimation of the LBHR/HBLR phenotypes for each patient and organ/pathology by obeying the following conditions: for part C (before exposure to EMF), if the I-intensity was found equal or higher than the average intensity, the phenotype was considered as HB. If the I-intensity was found lower than the average intensity the phenotype was considered LB; for part D (during exposure to EMF), if the I-intensity was found equal to or higher than the average intensity, the phenotype was considered HR. If the I-intensity was found lower than the average intensity, the phenotype was considered LR. From this process, two subsets (namely, 1′ and 2′) were deduced, gathering the LBHR and HBLR phenotypes, respectively (Table 1 and Table 2).
- Calculation of “null-responses ratios (NR): if I-intensities remained the same before (part C) and during the response to exposure to EMF (part D), the response was considered as NR.
- Calculation of the matching ratios (MR), reflecting whether the subsets 1′ and 2′ defined by the parts C and D questionnaire would correspond to the subsets 1 and 2 defined by the part A questionnaire, respectively (Table 1 and Table 2).

The data analysis of the I-histograms shown in Figure 3 reflected a great diversity of self-assessed responses according to the organ and the pathologies considered. The obtained with all the cerebral features while the lowest MR and the highest NR corresponded to the genito-urinary and the respiratory systems (Table 1 and Table 2; Figure 3). In addition, it appeared that some organs/pathologies may be associated with specific HBLR/LBHR phenotypes (Table 1): for example, the HBLR phenotype integrates more frequently fatigue and sleep disorders and slowing down of intellectual activity while the LBHR phenotype means of the self-assessed intensities of the symptoms before the exposure to EMF sources did not exceed 2, the lowest intensities concerning skin, genito-urinary, and cardiac and respiratory systems. The means of the self-assessed intensities of the symptoms during the exposure to EMF sources were found on average, 2.59 times higher than the corresponding values before exposure (Table 1). The highest self-assessed I-intensities of symptoms potentially induced by EMF were found higher than 4 and were obtained for cerebral features like headaches, tinnitus, slowing down of intellectual activity, fatigue, and sleep disorders while the lowest intensities (I ≈ 2.00) were observed for the genito-urinary and the respiratory systems (Table 1 and Table 2; Figure 3). By analyzing the MR and NR, the conclusions were similar: the highest MR and the lowest NR were also frequently composed of troubles in the cardiac and digestive system, troubles, mood instability and nervousness, headaches and tinnitus, and skin pathologies (Table 1).

Lastly, for each DEMETER donor, the respective frequency of LBHR, HBLR, and NR phenotypes was calculated from the 11 organ/pathologies proposed, and the major feature was directly attributed to the donor. The first conclusion emerging from this method was the relatively high value of the NR ratio (10/25 cases; 40%). The subsets 1′ and 2′ were found composed of 9/25 (36%) and 6/25 (24%) LBHR and HBLR phenotypes, respectively. Among these phenotyped subsets 1′ and 2′, only 5/15 cases (33%) matched with the subsets 1 and 2 defined from part A of the questionnaire (Table 2). It must be stressed here that to average all the values corresponding to each organ/pathology (while some of them are not necessarily representative of each phenotype) and to arbitrarily attribute the major LBHR/HBLR/NR feature to each patient may introduce some artifacts in the definition of the subset 1′ and 2′ by favoring NR.

### 2.2. Analysis of Biological Data from Skin Fibroblasts Derived from the DEMETER Donors

As stated in Materials and Methods, all the raw biological data from the skin fibroblasts derived from the DEMETER donors have been obtained before the opening of the sealed envelopes. It must be stressed that the self-assessment questionnaire data and the biological data were analyzed independently before performing inter-comparisons leading to the definitions of the different subsets.

#### 2.2.1. Spontaneous Data from the DEMETER Cohort

**Figure 4:**
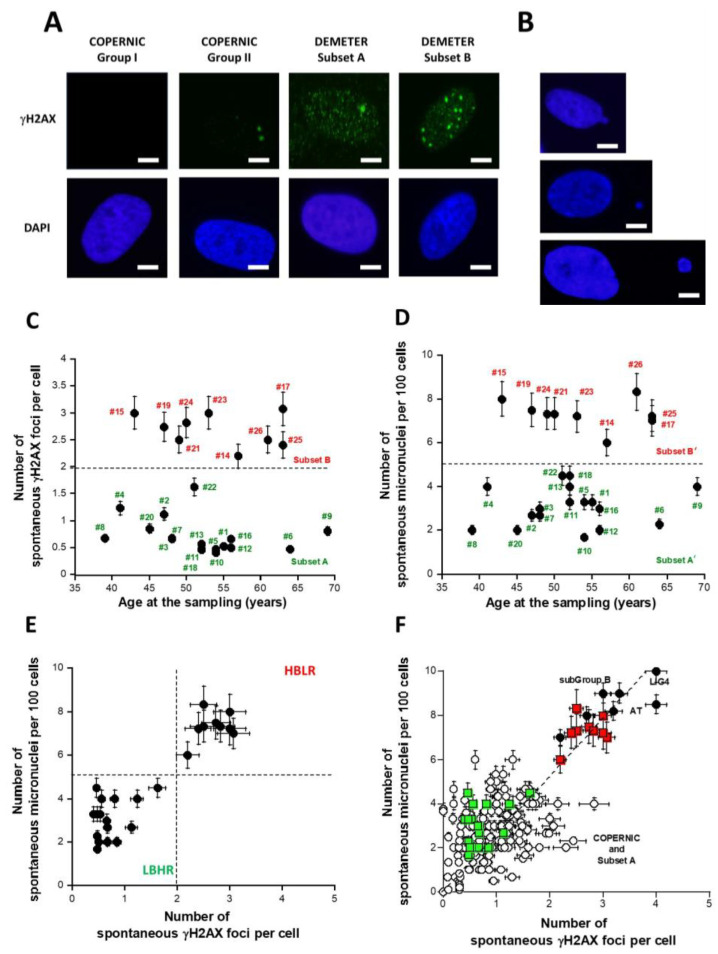
Number of spontaneous γH2AX foci per cell and micronuclei (MN) per 100 cells in fibroblasts from the DEMETER collection. Representative immunofluorescence images of spontaneous γH2AX foci (**A**) and micronuclei (**B**) from the indicated fibroblasts. The white bar corresponds to 5 µm. Spontaneous γH2AX foci data (**C**) and MN data (**D**) as a function of the age of DEMETER donors at the sampling. Each data is the mean ± SEM of at least three independent replicates. (**E**) The data shown in panel C were plotted against the corresponding data shown in panel D. Dashed line corresponds to limits defined in the text. Such a graph characterizes the LBHR and the HBLR phenotypes. Each data is the mean ± SEM of at least three independent replicates. (**F**) The 26 DEMETER γH2AX foci and MN data (squares) were plotted on the same graph as the 195 COPERNIC data (circles) published elsewhere [40]. Radioresistant controls and intermediate radiosensitivity COPERNIC data (open circles) and the hyper-radiosensitive *ATM*- and *LIG4*-mutated COPERNIC data (closed circles) were plotted with subset A (green squares) and B (red squares) DEMETER data. Each data is the mean ± SEM of at least three independent replicates. The dashed line represents the linear relationship between MN and γH2AX foci (y = 2.6x; r = 0.92).

The number of spontaneous DSB, key DNA damage, and the number of spontaneous micronuclei (MN), a major feature of the genomic instability, have been assessed in each DEMETER fibroblast cell line cultured at similar early passages. Spontaneous DSB was assessed by the anti-*γH2AX* immunofluorescence. The number of MN per 100 cells was scored on the same microscopic slides with the DAPI counterstaining (see Materials and Methods) [40] (Figure 4A,B).

The DEMETER fibroblasts showed on average 1.4 ± 0.2 spontaneous γH2AX foci suggesting an amount of spontaneous DSB significantly different from 0 (*p* < 0.001). When the number of γH2AX foci was plotted against the corresponding age of the donor, two distinct (*p* < 0.01) subsets of DEMETER donors appeared, namely the subsets A and B: the subset A whose cells showed less than 2 γH2AX foci (N = 17; mean: 0.71 ± 0.07 γH2AX foci) and the subset B whose cells showed more than 2 γH2AX foci per cell (N = 9; mean: 2.7 ± 0.1 γH2AX foci) (Figure 4C).

In parallel, the number of spontaneous MN was assessed in the DEMETER fibroblasts: the DEMETER fibroblasts showed 4.5 ± 0.4 spontaneous MN on average suggesting an amount of unrepaired chromosome breaks significantly different from 0 (*p* < 0.001). When the number of spontaneous MN was plotted against the corresponding age of the donor, two distinct (*p* < 0.01) subsets of donors appeared, namely the subsets A’ and B’: the subset A’ of donors whose cells showed less than 5 spontaneous MN per 100 cells (N = 17; mean: 3.0 ± 0.2 spontaneous MN per 100 cells) and the subset B’ of donors whose cells showed more than 5 spontaneous MN per 100 cells. (N = 9; mean: 7.3 ± 0.2 spontaneous MN per 100 cells) (Figure 4D).

Interestingly, the subsets A and A’ and B and B’ were composed of the same DEMETER donors, respectively (Figure 4C,D). Indeed, when the number of spontaneous MN and the corresponding number of spontaneous γH2AX foci were plotted together, data revealed that A = A’ and B = B’. Furthermore, all the data obeyed a linear function linking the two endpoints y = 2.6x; r = 0.92 (Figure 4E). Such a mathematical link was found in agreement with published data [40].

By considering the subsets 1 and 2 defined from the self-assessment questionnaire part B data, the average numbers of spontaneous γH2AX foci of each subset were found to be 1.43 ± 0.30 and 1.35 ± 0.27 γH2AX foci and the average numbers of spontaneous MN were found to be 4.60 ± 0.70 and 4.50 ± 0.55 MN, respectively: these values were not significantly different (*p* > 0.1), suggesting that the subsets 1 and 2 defined above cannot be discriminated by the number of spontaneous γH2AX foci or MN as endpoint. By contrast, it must be stressed that 9/11 (81.8%) and 7/15 (46.6%) subsets A and B donors belong to subsets 1 and 2, respectively. The rest of the donors (2/11 (18.1%) and 8/15 (53.3%)), respectively, were dispatched in the other subset.

The relationship between MN and γH2AX foci was in agreement with data published previously with the COPERNIC collection [40]. In order to better document the relevance of such findings, we have plotted the 26 DEMETER data on the same graph as the 195 COPERNIC data published elsewhere [40] (Figure 4F). Such a relationship between spontaneous MN and γH2AX foci appeared to be similar (*p* > 0.6) to that obtained previously with the LBHR/HBLR phenotypes shown in Figure 4E, supporting the quantitative relevance of our findings [40]. Interestingly, the levels of spontaneous DSB and MN observed in DEMETER fibroblasts corresponding to a HBLR phenotype were found similar to those observed in fibroblasts deriving from the highest hyper-radiosensitivity syndromes, namely ataxia telangiectasia (AT), caused by *ATM* mutations and LIG4 syndrome, caused by *LIG4* mutations [40]. At this step, it must be stressed that: (1) electrosensitivity has not been described in AT and LIG4 syndrome patients; (2) reciprocally, the HBLR phenotype is strongly different from that of *ATM*- and *LIG4*-mutated patients [40].

#### 2.2.2. Perinuclear Crowns of the ATM Protein in DEMETER Fibroblasts

**Figure 5:**
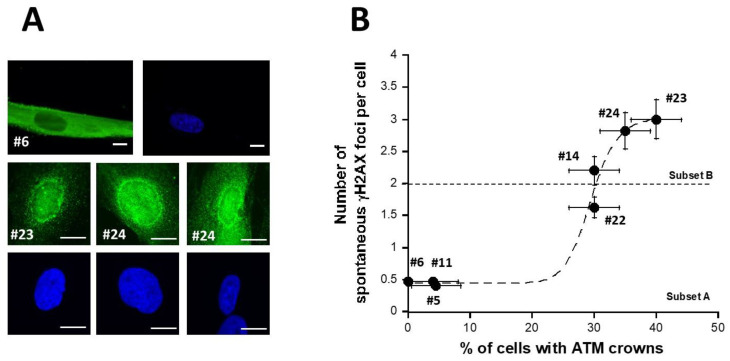
pATM perinuclear crowns in DEMETER cells. (**A**) Representative immunofluorescence images showing pATM perinuclear crowns (green) in the indicated DEMETER subset B fibroblasts (lower panel) or absence of pATM perinuclear crowns in the indicated DEMETER fibroblasts subset A (upper panel). All cells were cultures at passages 30–40. Nuclei were counterstained with DAPI (blue). White bar represents 10 µm. (**B**) Number of spontaneous γH2AX foci per cell as a function of the percentage of cells with pATM crowns. Each data from the indicated subset A and B DEMETER fibroblast data is the mean ± SEM of at least two independent replicates. Data were fitted to a sigmoidal function (dashed line) (r = 0.988).

Recently, we have published 2 studies showing that permanent genotoxic stress or a low dose rate of gamma-irradiation progressively monomerizes the dimeric forms of the ATM protein that diffuse to the nucleus [42,43]. As a result, around the nucleus, the too-high local concentration of ATM monomers facilitates the redimerization of ATM, which triggers the formation of a perinuclear ATM crown. Such perinuclear ATM crowns have been shown to be a specific biomarker of the accelerated aging phenomenon [42,43]. Since the DEMETER fibroblasts associated with the HBLR phenotype elicited spectacular levels of spontaneous DNA breaks (Figure 4), we examined whether DEMETER fibroblasts cultured at high passages elicited perinuclear ATM crowns. For practical reasons, the ATM crowns assay was not applied to all 26 DEMETER cells but to 7 representative cell lines. Only the DEMETER fibroblasts of the subset B tested showed perinuclear ATM crowns at cell passages 30–40. The number of spontaneous γH2AX foci appeared to be a sigmoidal function of the percentage of the perinuclear ATM crowns (Figure 5). These findings suggested that the formation of perinuclear ATM crowns requires a threshold of more than 2 γH2AX foci per cell (Figure 5). Altogether, these data are consistent with the fact that the HBLR phenotype may be associated with accelerated aging [42,43].

#### 2.2.3. X-Rays-Induced Data from the DEMETER Cohort

The formation of the perinuclear ATM crowns is generally associated with a delay in the ATM nucleoshuttling in response to permanent stress [42,43]. Since 2014, we have developed a mechanistic model to explain the individual response to oxidative stress, notably ionizing radiation. Briefly, in proportional response to oxidative stress, some cytoplasmic ATM dimers monomerize and diffuse into the nucleus: the ATM monomers recognize the stress-induced DSB by phosphorylating the X-variant histones H2AX surrounding the DSB sites, which results in the formation of γH2AX foci. Once DNA ends are ligated, ATM monomers dimerize and form the pATM foci [44]. Such a process can be delayed by the cytoplasmic over-expression of some ATM substrates, called X-proteins, that form ATM-X protein complexes in the cytoplasm. As a result, the flux of ATM monomers diffusing to the nucleus decreases and leads to a lack of DSB recognition which causes toxicity (when DSB are unrepaired), cancer proneness, and radiosusceptibility (when DSB are misrepaired) or accelerated aging and radiodegeneration (when DSB are tolerated and cumulate in the nucleus). This is notably the case after irradiation with a delay of the radiation-induced ATM nucleoshuttling (RIANS) [44]. In the frame of the RIANS model, 3 groups of radiosensitivity were defined [44]: group I, radioresistance, rapid RIANS; group II, intermediate radiosensitivity, delayed RIANS; group IIIa, hyper-radiosensitivity, gross ATM kinase defects; group IIIb, hyper-radiosensitivity, normal ATM kinase but gross DSB repair defect [44]. In order to examine the RIANS status of the DEMETER fibroblasts, similarly to the COPERNIC ones, cells were exposed to 2 Gy X-rays, and the kinetics of γH2AX and pATM foci were analyzed.

**Figure 6:**
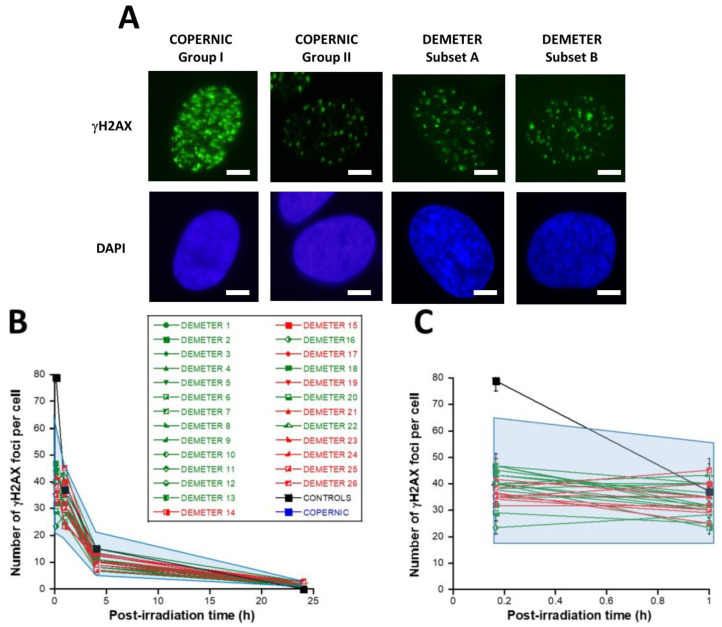
Kinetics of appearance/disappearance of γH2AX foci. (**A**) Representative immunofluorescence images of γH2AX foci after 2 Gy followed by 10 min post-irradiation. The white bar corresponds to 5 µm. (**B**) The number of γH2AX foci after 2 Gy X-rays followed by the indicated post-irradiation time (h) was plotted against post-irradiation time. Each plot represents the mean ± SEM of 3 replicates. The control and the subset A and B data were illustrated by black, green, and red symbols, respectively. The blue confidence zone summarizes where the COPERNIC data can be found. For each post-irradiation time, the control data are represented by the mean of the 11 pooled control cell lines data defined elsewhere [40,41]. (**C**) Panel (**C**) is a zoom over the first hour post-irradiation of panel (**B**).

First, let’s describe the kinetics of γH2AX foci usually observed in the radioresistant control COPERNIC fibroblasts (Figure 6A). In the radioresistant control COPERNIC fibroblasts, the number of γH2AX foci scored 10 min after 2 Gy was 79 ± 4 per cell, in agreement with the very documented value of 37 ± 4 per Gy per cell [40,41] (Figure 6B,C). The number of γH2AX foci thereafter decreased with time to reach its minimal amount at 24 h post-irradiation. For the radioresistant control fibroblasts, the number of γH2AX foci assessed at 24 h post-irradiation was not different from zero (*p* < 0.01) in good agreement with a number of published data (Table 3).

In the DEMETER fibroblasts, the number of γH2AX foci scored 10 min after 2 Gy was found on average, 2.05 lower than controls (*p* < 0.001) [23.33–46.66], suggesting an impairment of the DSB recognition step (Table 3) (Figure 6B,C). Interestingly, by calculating the average numbers of γH2AX foci scored 10 min after 2 Gy for each subset 1 and 2, it appeared that both values were not found different (*p* > 0.3). Conversely, the average numbers of γH2AX foci scored 10 min after 2 Gy of each for subset A and B were found significantly different (*p* < 0.05), suggesting that the phenotype HBLR is associated with the most impaired DSB recognition process (Table 3). By analyzing the number of γH2AX foci scored 24 h after 2 Gy, similar conclusions were reached: the average numbers of residual γH2AX foci of subsets 1 and 2 were not found different (*p* > 0.5) while those of subsets A and B were significantly different (*p* < 0.05), suggesting that the phenotype HBLR is also associated with an impaired DSB repair process when observed with anti-*γH2AX* immunofluorescence (Table 3; Figure 6).

**Figure 7:**
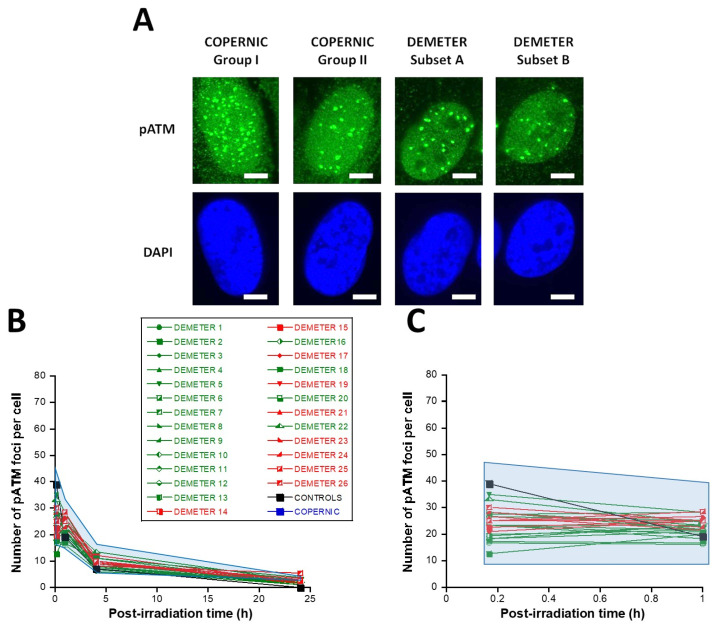
Kinetics of appearance/disappearance of pATM foci. (**A**) Representative immunofluorescence images of pATM foci after 2 Gy followed by 10 min post-irradiation. The white bar corresponds to 5 µm. (**B**) The number of pATM foci after 2 Gy X-rays followed by the indicated post-irradiation time (h) was plotted against post-irradiation time. Each plot represents the mean ± SEM of 3 replicates. The control and the subset A and B data were illustrated by black, green, and red symbols, respectively. The blue confidence zone summarizes where the COPERNIC data can be found. The control data are, for each post-irradiation time, the mean of the 11 pooled control cell lines data defined elsewhere [40,41]. (**C**) Panel (**C**) is a zoom over the first hour post-irradiation of panel B.

In the DEMETER fibroblasts, the number of pATM foci scored 10 min after 2 Gy was found 1.68 lower than controls (*p* < 0.01) [12.5–35], suggesting an impairment of the DSB recognition step (Table 4; Figure 7). Interestingly, by recalculating the average numbers of pATM foci scored 10 min after 2 Gy for each subset 1 and 2, it appeared that both values were not found different (*p* > 0.1). The same conclusions were reached with the values corresponding to the subsets A and B (*p* = 0.08). By analyzing the number of pATM foci scored 24 h after 2 Gy, similar conclusions were reached: the average numbers of residual pATM foci of subsets 1 and 2 and of subsets A and B were not found different (*p* > 0.5). The absence of significant differences was probably due to the fact that all the pATM values were much lower than their γH2AX counterparts (Table 4; Figure 7B,C).

In addition, the fact that the number of γH2AX and pATM foci assessed 10 min post-irradiation was found lower in DEMETER fibroblasts than in the COPERNIC Group I ones, strongly suggests the existence of X-proteins, ATM substrates, that may be overexpressed in the cytoplasm of DEMETER fibroblasts, leading to an impairment of the DSB recognition. However, further investigations are needed to identify such X-proteins, inasmuch as they may be specific to each DEMETER patient.

Lastly, in the frame of the RIANS model, we have also shown that the MRE11 endonuclease may depend on the ATM nucleoshuttling. Indeed, after irradiation, the phosphorylation of MRE11 by ATM monomers disrupts the nuclease activity of MRE11 and is responsible for the formation of nuclear MRE11 foci. If MRE11 foci appear lately after irradiation (e.g., 24 h post-irradiation), a number of DNA breaks cumulate in the nucleus. Late MRE11 foci have been generally associated with aging syndromes [45]. By contrast, early MRE11 foci (i.e., occurring in the first hour post-irradiation) have revealed a sudden DNA break production, generally associated with the hyper-recombination phenomenon that is a very common feature of cancer syndrome [45].

**Figure 8:**
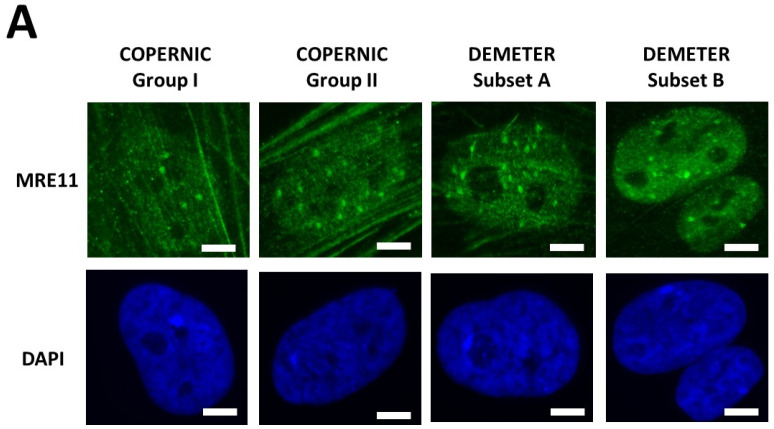

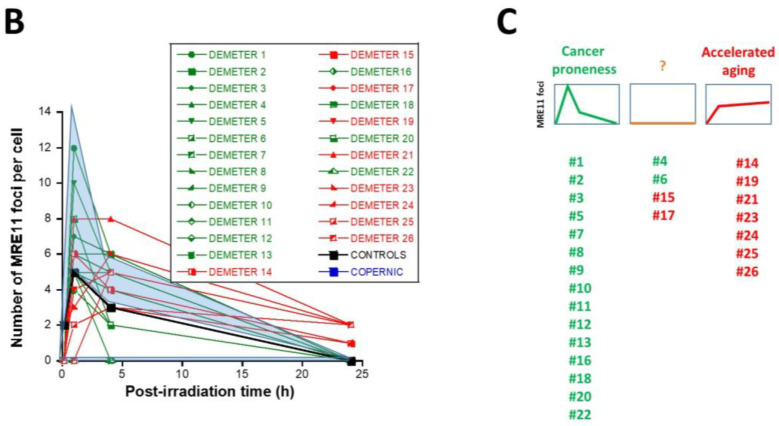
Kinetics of appearance/disappearance of MRE11 foci. (**A**) Representative immunofluorescence images of MRE11 foci after 2 Gy followed by 1 h post-irradiation. The white bar corresponds to 5 µm. (**B**) The number of MRE11 foci after 2 Gy X-rays followed by the indicated post-irradiation time (h) was plotted against post-irradiation time. Each plot represents the mean ± SEM of 3 replicates. The control and the subset A and B data were illustrated by black, green, and red symbols, respectively. The blue confidence zone summarizes where the COPERNIC data can be found. The control data are, for each post-irradiation time, the mean of the 11 pooled control cell lines data defined elsewhere [40,41]. (**C**) Panel (**C**) is a schematic representation of the 3 shape types of the MRE11 foci kinetics and details the corresponding DEMETER cell lines that elicited the given shape type.

In agreement with our previous publications, the radioresistant control fibroblasts from apparently healthy donors elicited a low number of MRE11 foci that increased from 1 h to 4 h post-irradiation, when the maximal number of MRE11 was reached and progressively decreased up to zero foci at 24 h post-irradiation [45] (Figure 8). From our previous data, three shapes of MRE11 foci kinetics have been observed:

- Type 1: an Euler’s gamma function shape with an early peak and a large decrease from 4 h to 24 h post-irradiation. The type 1-shape of MRE11 foci kinetics has been generally observed in cells deriving from cancer syndromes like neurofibromatosis type I syndrome [46].
- Type 2: a “zero” function reflecting the absence of MRE11 foci in the [10 min–24 h] post-irradiation range. The type 2-shape of MRE11 kinetics generally reflects impaired or absent MRE11 foci as observed in syndromes caused by a mutated helicase (Bloom’s syndrome or Werner syndrome) or those caused by mutated protein upstream MRE11 function like ataxia telangiectasia or Nijmegen syndrome [45].
- Type 3: a curvilinear shape with a progressive increase of the number of MRE11 foci and a horizontal or a low-decrease-rated plateau. The type 3-shape of MRE11 kinetics has been generally observed in cells deriving from aging syndromes [45].

In the DEMETER fibroblasts, the three types of MRE11 kinetics were observed. However, by considering the subsets A and B as the reference subsets and the early/late MRE11 foci as the reference endpoint, 15/18 (83.3%) cell lines from subset A showed a type-1 shape of MRE11 foci kinetics and did not show a type-3 one. Similarly, 7/9 (77.7%) cell lines from subset B showed a type-3 shape of MRE11 foci kinetics and did not show a type-1 one. All these data suggest that MRE11 foci provided a good sensitivity score to discriminate subset A and subset B and their specific associated phenotypes. Hence, our findings showed that the LBHR and HBLR phenotypes are associated with early and late MRE11 foci, respectively (Table 5) (Figure 8).

#### 2.2.4. H_2_O_2_-Induced Data from the DEMETER Cohort

**Figure 9:**
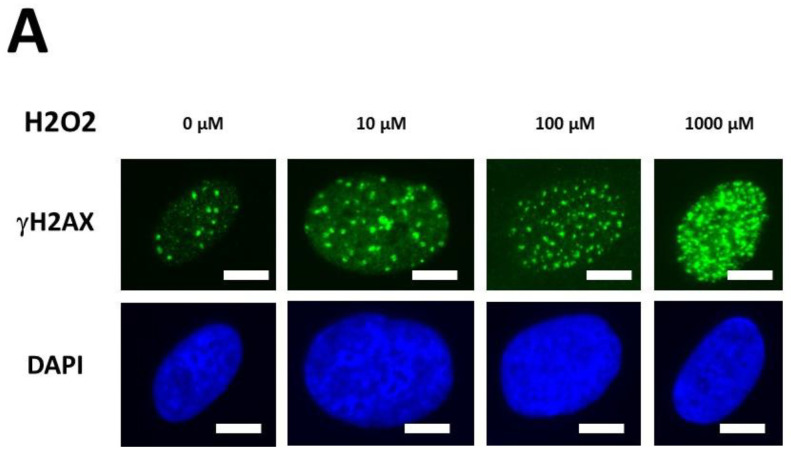

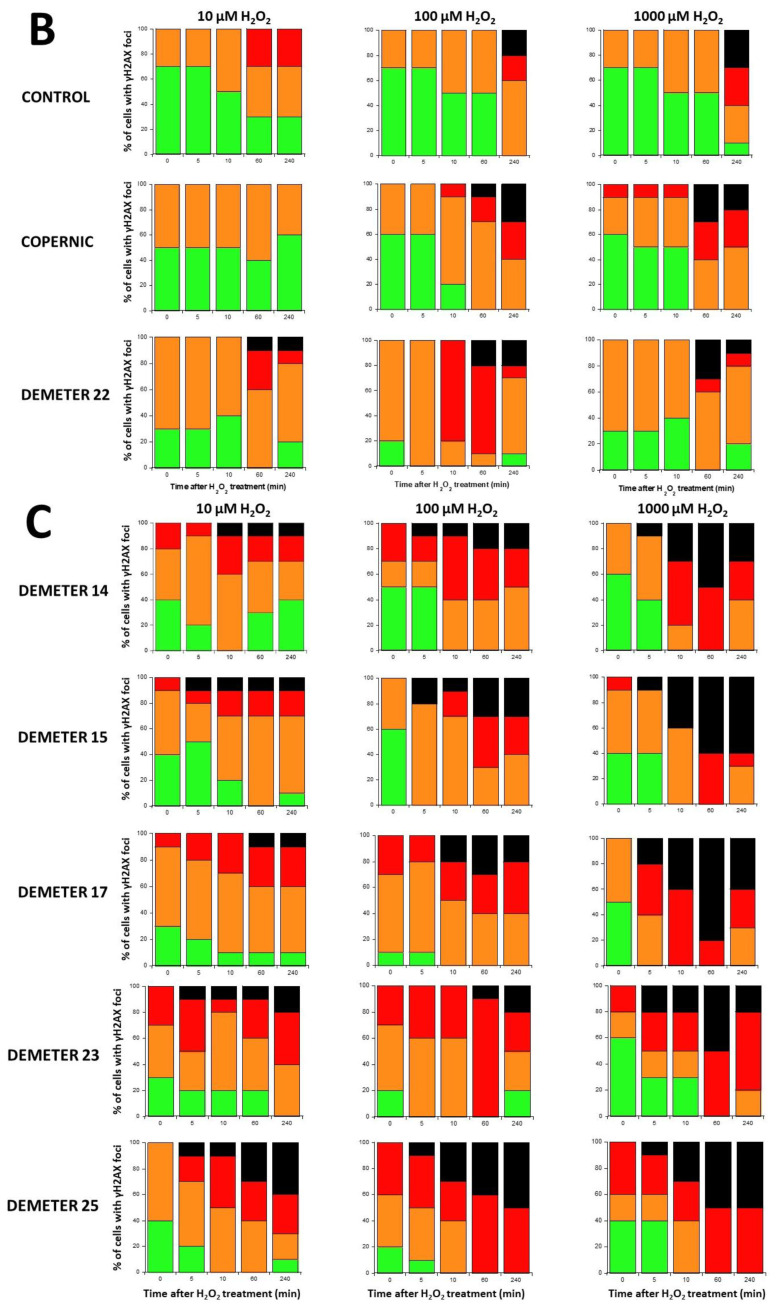
γH2AX foci data after H_2_O_2_ treatment. (**A**) Representative immunofluorescence images of γH2AX foci after the indicated H_2_O_2_ treatment followed by 10 min in representative DEMETER subset B fibroblasts. The white bar corresponds to 5 µm. (**B**,**C**). Histograms representing the percentage of cells with γH2AX foci as a function of the time after H_2_O_2_ treatment. The indicated final concentration of H_2_O_2_ was added directly to the cells for 30 min and a post-stress time was applied. After anti-*γH2AX* immunofluorescence procedure, four categories of cells were observed: cells without γH2AX foci (green), cells with 1–15 γH2AX foci (orange), cells with 16–30 foci (red) and highly damaged cells (HDC) (black). Each percentage is the mean of two independent experiments, at least. (**B**). Data presented: one representative COPERNIC Group I radioresistant control cell line, one representative COPERNIC Group II radiosensitive cell line, and DEMETER 22 cell line (belonging to the subset A). (**C**). Data from the indicated DEMETER cell lines belonging to the subset (**B**).

The kinetics of the MRE11 nuclease activity in the DEMETER fibroblasts raised the question of the management of both DNA single-strand breaks (SSB) and DSB. Indeed, the presence of SSB surrounding DSB sites may increase the severity of DSB through chromatin decondensation but also trigger the formation of highly damaged cells (HDC), which has been documented recently as a final step of perinuclear pATM crown [43]. Hence, to investigate further the molecular features of DEMETER fibroblasts, we treated cells with hydrogen peroxide (H_2_O_2_), a very documented SSB and DSB inducer [47]. Three H_2_O_2_ concentrations (10, 100, 1000 µM) have been applied to representative DEMETER cell lines for 30 min to induce SSB only, a mixture of SSB and DSB and DSB only, respectively [47]. The number of DSB per cell was assessed by anti-*γH2AX* immunofluorescence. Again, for practical reasons, the H_2_O_2_ assay was not applied to all the DEMETER cells but to 6 representative ones (Figure 9A).

**Figure 10:**
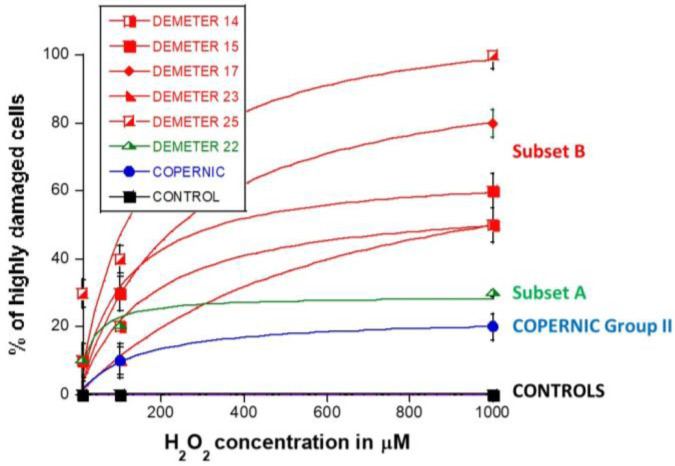
Percentage of HDC in H_2_O_2_-treated DEMETER fibroblasts as a function of H_2_O_2_ concentration. The data shown here are similar to the 240 min data shown in Figure 9. Each plot represents the mean ± SEM of two independent replicates. Data were fitted to the Michaelis-Menten equation y = ax/(b + x). See resulting values of adjustable parameters and correlation coefficients in Table 6.

Four categories of cells were observed: cells without γH2AX foci (green), cells with 1–15 γH2AX foci (orange), and cells with 16–30 foci (red) and HDC (black). The radioresistant control cell lines showed a low amount of γH2AX foci and a slow progression towards the formation of HDC that were observed only after 240 min post-treatment for only 100 and 1000 µM H_2_O_2_ (Figure 9B,C). In COPERNIC cells, the formation of HDC began 60 min post-treatment. However, like controls, HDC appeared only at the two highest H_2_O_2_ concentrations (Figure 9B). Interestingly, the DEMETER 22 data (subset A) were found similar to the COPERNIC with the notable exception that some HDC appeared already at 60 min after 10 µM H_2_O_2_. By contrast, all the DEMETER cell lines belonging to the subset B tested (Figure 9B) elicited an early and abundant production of HDC suggesting that the high background observed spontaneously in these cells may help in the formation of HDC likely via a chromatin decondensation caused by the formation of numerous SSB. However, there was no presence of spontaneous HDC: 5 min post-treatment is required, at least, for the formation of HDC, suggesting that both SSB and DSB repair are required for initiating this process (Figure 9B). In order to better illustrate this molecular feature that would be specific to the subset B, the percentage of HDC assessed at 240 min post-treatment was plotted against the concentration of µM H_2_O_2_ (Table 6) (Figure 10).

The percentage of HDC as a function of the H_2_O_2_ concentration was shown to obey a curvilinear function (like the Michaelis-Menten function) whose adjustable parameters provide interesting properties: in the fitting formula: y = ax/(b + x), the parameter a indicated the maximal percentage of HDC (value at the plateau) reachable and the parameter b indicated the H_2_O_2_ concentration reached when half of this maximal percentage of HDC is reached. Hence, it appeared clear that the maximal percentage of HDC was significantly lower in COPERNIC group II and in DEMETER subset A cells than in DEMETER subset B cells (*p* < 0.001), suggesting that the formation of HDC cells after exposure to H_2_O_2_ may be used to discriminate both subset A and B but not necessarily COPERNIC Group II and DEMETER subset A (Figure 10).

**Figure 11:**
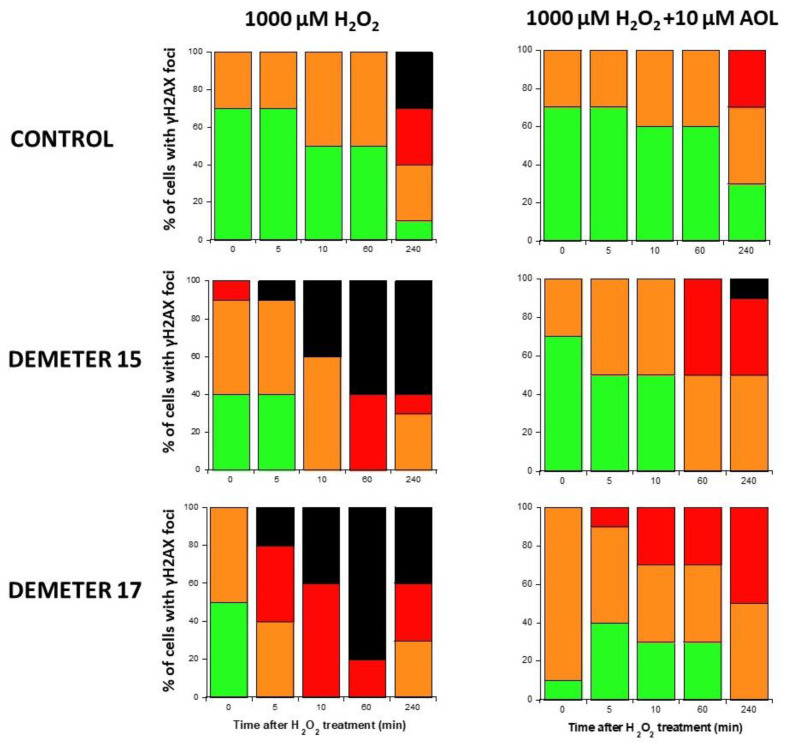
Histograms representing the percentage of cells with γH2AX foci as a function of the time after H_2_O_2_ treatment. The indicated final concentration of H_2_O_2_ was added directly to the cells for 30 min and a post-stress time was applied. A pre-treatment with 10 μM AOL has been applied to cells for 24 h before the H_2_O_2_ treatment when indicated. After anti-*γH2AX* immunofluorescence procedure, four categories of cells were observed: cells without γH2AX foci (green), cells with 1–15 γH2AX foci (orange), cells with 16–30 foci (red) and highly damaged cells (HDC) (black). Each percentage is the mean of two independent experiments, at least.

Since our findings suggested that the DEMETER subset B fibroblasts involve high levels of SSB and DSB and therefore high levels of oxidative stress, we examined whether treatment with a documented anti-oxidative agent may affect the formation of HDC. Anetholtrithione (AOL) was chosen as an anti-oxidative agent because of its radioprotection capacities [48,49,50]. By focusing only on two DEMETER subset B fibroblast cell lines, namely #15 and #17, Figure 11 shows that a 24 h pre-treatment with 10 μM AOL was sufficient to reduce significantly the formation of HDC cells in the DEMETER subset B fibroblasts tested. These findings reinforce the importance of permanent oxidative stress in the process of formation of HDC (Figure 11). Further investigations are needed to evaluate the actual interest of an anti-oxidative approach as a prevention or therapy for EHS and its effect on each symptom described.

## 3. Discussion

### 3.1. Some Molecular Features to Better Understand EHS?

The DEMETER study was based on a cohort of 26 adult volunteers self-diagnosed as suffering from EHS. They have all freely agreed to fill in a self-assessment questionnaire and to provide one skin biopsy to compose a unique collection of primary fibroblasts, in full respect of the current national ethical regulations. Whether from questionnaire or fibroblast data, two subsets of DEMETER donors, eliciting LBHR or HBLR phenotypes each, have been defined with different endpoints. All these steps are summarized in Table 7 and Table 8:

- part A of the questionnaire (symptoms after exposure to different sources of EMF) suggested 2 subsets of donors (subsets 1 and 2) whose LBHR and HBLR phenotypes were defined quantitatively by the self-assessed intensities symptoms linked to the different sources of EMF.
- from part B of the questionnaire (symptoms after exposure to other sources than EMF), the HBLR phenotype was suggested to be associated with some immunodeficiency.
- in parts C and D of the questionnaire (symptoms before or during exposure to EMF), the highest self-assessed intensities of symptoms were obtained for cerebral features like headaches, tinnitus, slowing down of intellectual activity, fatigue, and sleep disorders.
- parts C and D of the questionnaire also suggested 2 subsets of donors (namely subsets 1′ and 2′) whose definition was based on the self-assessed intensities of symptoms before and during the exposure to EMF, by considering different organs or pathologies. However, the too-frequent null responses did not permit us to obtain a sufficiently high matching ratio (on average, 58%, (Table 7)) with the subsets 1 and 2 defined before.
- parts C and D of the questionnaire also revealed that the HBLR phenotype was particularly associated with fatigue, sleep disorders, and the decrease of intellectual capacity while the LBHR phenotype was particularly associated with impairment of cardiac and digestive systems, mood instability, nervousness, headaches, tinnitus and skin reactions.
- by taking the yield of spontaneous DSB or MN as endpoints, two series of subsets of DEMETER volunteers, namely A and B, and A’ and B’ were defined, respectively. Our data showed that A and A’, on one hand, and B and B’, on another hand, were composed of the same DEMETER donors. In addition, each of the two subsets A and B was found included in one of the two subsets 1 and 2 described above with a matching ratio that did not exceed 64% identity (Table 7).
- by applying X-rays, all the DEMETER cell lines (26/26) were characterized by delayed RIANS like the patients of the COPERNIC Group II (who do not suffer from EHS symptoms). These findings suggest that EHS may be systematically linked to delayed RIANS but this statement is not reciprocal. It also means that, like the COPERNIC Group II cells, some X-proteins, ATM substrates, should be overexpressed in the cytoplasm in all the DEMETER cells. Such X-proteins can be specific to each DEMETER individual and its overexpression may result from a heterozygous mutation [44].
- the molecular definition of the subsets A and B was found consolidated by other biomarkers: subset A was associated with an early formation of radiation-induced MRE11 foci while subset B was associated with both spontaneous formation of perinuclear pATM crowns and late formation of radiation-induced MRE11 (92.3% identity). From our documented data published elsewhere [45], these findings suggest that subset A was composed of donors with a high risk of cancer while subset B was composed of donors with a high risk of accelerated aging.
- by applying H_2_O_2_ treatment to DEMETER cells, SSB and DSB may lead to the formation of HDC, that were shown to be the final step of perinuclear pATM crowns [42]. The differences observed with H_2_O_2_ treatment between subset B (HBLR phenotype) and controls significantly decreased with the application of an anti-oxidative treatment, reinforcing again the model that EHS may be related to the management of SSB and/or DSB.

### 3.2. The Current Limits of a Self-Assessment Questionnaire

Self-assessment questionnaires have been currently used for the EHS research [51,52,53]. Among the diversity of responses possible in a self-assessment questionnaire, literature has suggested the existence of a number of different subsets of volunteers. Particularly, some specific clinical phenotypes have been evoked [17,24,25,51,52,53]. However, no biological data were associated with the definition of any subset yet.

In addition, there are a number of biases associated with any self-assessment questionnaire that can affect the validity of responses and give a distorted picture of a person’s perceptions or behaviors [54,55]. Here, the question of biases is inasmuch important that the number of DEMETER volunteers is low (n = 26). Among the potential biases, the bias of social desirability is of importance: it may result in an adjustment of the responses of volunteers to better reach what they perceive to be socially acceptable or expected, instead of responding honestly [54,55]. For example, they might downplay negative behaviors or, conversely, exaggerate positive behaviors. Even if it is difficult to define the countermeasures against such a bias, by calculating the average self-assessed intensities for the C and D parts of the questionnaire for each DEMETER donor, we have verified that all the data obey the cumulative Gaussian function (Figure S1; Supplementary Data), which reduces the possibility that some donors exaggerate systematically their responses and influence the analysis of the whole data.

Similarly, since DEMETER volunteers are members of the same association (here, namely Electrosensibles de France/PRIARTEM), one can evoke also bias, the “group effect”, that can lead to a certain uniformity in the responses. Again, the cumulative Gaussian distribution of responses (Figure S1; Supplementary Data) and the existence of two distinct phenotypes that are in coherence with biological data are not consistent with uniform responses. However, further investigations are needed to better consider each type of bias and to apply countermeasures when possible.

Lastly, we are all aware that the size of the DEMETER cohort is low. However, one must also consider the difficulty for EHS patients to move, travel, and leave their homes where they often have protection against waves. In addition, reception in a medical environment represents a real challenge for some EHS patients. However, it should be noted that the finding that 26/26 DEMETER patients show delayed RIANS suggests that a larger cohort would not have drastically changed this final conclusion.

### 3.3. The Spontaneous Levels of DNA Breaks: A First Prerequisite for EHS?

The presence of spontaneous SSB, DSB, or MN in DEMETER cells likely reflects endogenous oxidative stress and significant genomic instability [56,57,58]. Few research groups have investigated the management of oxidative stress in cells from EHS individuals [32,59]: (1) maybe because of the paradigm that EMF cannot directly deliver energy sufficient to ionize and to break DNA (see above); (2) because the access to cell lines provided from EHS individuals is difficult in practice. However, some groups have performed investigations on blood cells but generally as dosages of proteins involved in the response to oxidative stress and more rarely with functional assays [32,60,61]. While it must be stressed that there is a plethora of biomarkers and assays related to the molecular and oxidative stress response, existing studies did not lead to a clear conclusion [32,60,61]. The collection of DEMETER fibroblasts was therefore an actual opportunity to apply the biomarkers of the RIANS model which is a very documented mechanistic model validated in a number of situations of stress [44,62,63]. Hence, by analyzing both DEMETER and COPERNIC fibroblast cell lines data with anti-*γH2AX* immunofluorescence, the number of spontaneous DSB did not exceed 2 γH2AX foci per cell to the notable exception of the DEMETER subset B, and the COPERNIC (group III) hyper-radiosensitive cell lines like those providing from ataxia telangiectasia or LIG4 syndrome patients (Figure 4). The same conclusion was reached with MN with a yield of 5 spontaneous MN per 100 cells (Figure 4). In fact, these conclusions agreed with the hypothesis that cells cannot survive with a certain degree of DNA or chromosome breakage [40,64]. However, the DEMETER subset B donors do not show the same clinical features as the hyper-radiosensitivity cases (i.e., high risk of leukemia, degeneration of Purkinje cells, hyper-radiotoxicity, immunodeficiency, and low life span) [65]. Conversely, no EHS was reported for *ATM*- and *LIG4*-mutated children and the series of symptoms observed in DEMETER subset B appeared to be much In fact, the same average number of DSB or MN was not dispatched in the same way in both cases: in hyper-radiosensitive fibroblasts, DSB and MN are equally dispatched in all the cells while in DEMETER subset B fibroblasts, such amount is generally due to a subpopulation of cells showing tens DSB: removing such specific subpopulation from the calculations will lead to values comparable to DEMETER subset A (Figure S2, Supplementary Data). Such specific features are likely due to the presence of cells that will lead to pATM crowns and HDC cells and may explain why, with the same amount of spontaneous DSB and MN, the clinical features of DEMETER subset B and COPERNIC group III donors are different.

**Figure 12:**
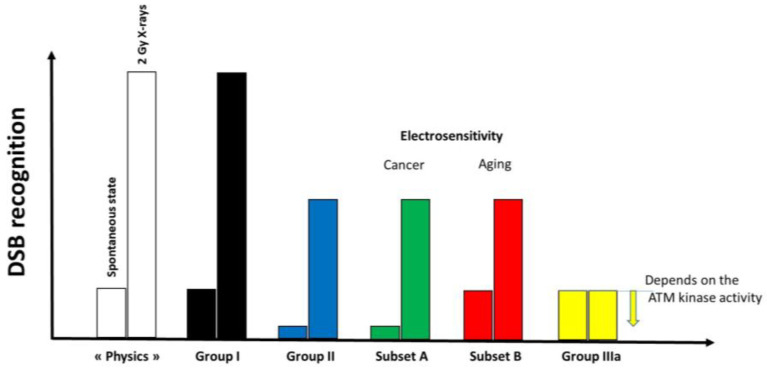
Schematic illustration of the DSB recognition according to the group of radiosensitivity and electrosensitivity. In the radioresistant group I cells, all the DSB induced are recognized. At the spontaneous state, the amount of DSB recognized is not complete in COPERNIC group II and DEMETER subset A cells while it is in DEMETER subset B cells. Conversely, for a larger amount of DSB like after an induction of 2 Gy X-rays, only 50% are recognized. In hyper-radiosensitive COPERNIC group III cells, the level of DSB recognition is lower than in group II cells but depends on the ATM kinase activity.

Since γH2AX foci were observed at the spontaneous state, it means that the ATM kinase was able to phosphorylate H2AX at the DSB sites and therefore to recognize spontaneous DSB. However, the same number of γH2AX foci may correspond to different amounts of “physical” DSB according to the DSB recognition rate (e.g., 4 physical DSB may correspond to 4 γH2AX foci if the DSB recognition is complete while it may correspond to 2 γH2AX foci if DSB recognition rate is 50%). Interestingly, the data from X-ray irradiation showed that more than 50% of radiation-induced DSBs are not recognized by ATM. Hence, the nuclear ATM kinase activity and the DSB recognition rate may depend on the amount of DSB. Interestingly, with regard to the DEMETER subset A, the spontaneous rates of DSB and MN are similar to those observed with COPERNIC Group II cells, suggesting a similar yield of “physical” DSB. The same conclusion can be reached with DEMETER subset B and COPERNIC group III cells. Hence, the link between the amount of “physical” DSB and the amount of the DSB recognized by the ATM-dependent pathway may differ according to the subset and the phenotype observed. Hence, one may conclude that (Figure 12):

- at a spontaneous rate, the DSB recognition rate is close to 100% because the number of DSBs is relatively low. However, the permanent stress may be higher in DEMETER subset B cells than in subset A ones, as the phenotype HB suggests, because of an impairment in the management of oxidative stress.
- after 2 Gy X-ray treatment, the DSB recognition rate is 50% for all the COPERNIC Group II and DEMETER cells, whatever the subset. In these conditions, the response of DEMETER Subset B cells is closer than that of COPERNIC Group II than COPERNIC Group III.

While a high spontaneous rate of DSB or MN is not necessarily specific to EHS, the DEMETER subset B fibroblasts may elicit higher endogenous stress with more DSB per cell (HB phenotype) than in subset A ones (LB phenotype), suggesting a stronger impairment in the management of the SSB and/or DSB and in their repair. These findings are consistent with the reactions of immunodeficiency reported specifically in this subset but also with the efficiency of the anti-oxidative drug after an H_2_O_2_ treatment.

### 3.4. EHS May Be Associated with a Delayed RIANS

The radiobiological characterization performed in the present report strongly suggests that all the DEMETER fibroblasts showed delayed RIANS. Even if the DEMETER cohort is low (n = 26), such a statement cannot be the result of a coincidence considering the biological and mathematical constraints linked to delayed RIANS [40]. Such RIANS status means that, if DEMETER donors were treated with radiotherapy for cancer, they may be at high risk of adverse tissue reactions post-treatment [40]. In our previous reports, the mathematical link between the maximal yield of pATM foci and the severity grade of the post-radiotherapy reactions (Common Terminology Criteria for Adverse events, CTCAE) has been very documented thanks to the COPERNIC collection: every 10 less pATM foci, the severity CTCAE grade increases by one additional unit [40,41]. From the findings presented here, the anti-*pATM* immunofluorescence data on X-rays irradiated cells suggest that DEMETER donors may be at risk of a grade 2–4 tissue reaction post-radiotherapy (like any COPERNIC group II patients), which represents non-negligible secondary effects. Interestingly, numerous symptoms cited for EHS belong to the list of the CTCAE grade 1 reactions. Our published data have shown that grade 1 corresponds to the lack of about 10 pATM foci per cell, i.e., the equivalent of an X-ray dose of 0.5 Gy. Further investigations are therefore needed to verify whether an EMF exposure of cells from EHS individuals may be equivalent to such an X-ray dose.

### 3.5. Two Subsets of EHS?

In addition to the delayed RIANS, the DEMETER fibroblasts show two subsets sharing LBHR and HBLR phenotypes whose definition depends on the endpoint considered (Table 8). We have previously shown that delayed RIANS may be caused by the overexpression of ATM substrates called X-proteins (see Results) [44]. In response to any oxidative stress, cytoplasmic ATM dimers monomerize and diffuse to the nucleus. The heterozygous mutations generally observed in group II cells are associated with subcellular relocalization of X-proteins and/or overexpression, which facilitates the formation of complexes between ATM and X-proteins in the cytoplasm [44]. Hence, these resulting ATM-X protein complexes prevent or delay the nucleoshuttling of ATM required for complete DSB recognition. Some X-proteins may be spontaneously induced or induced by ionizing radiation [44,66]. Can EHS be associated with an EMF-induced over-expression of X-proteins that would be specific to each subset? By reviewing the literature, it appears unlikely that, in non-thermal conditions, some proteins show a significant EMF-dependent expression [67,68]. By contrast, post-translational modifications (PTM) that may lead to aberrant subcellular localization, facilitated protein-protein interactions or repulsions, phosphorylations, and methylations have been observed in response to EMF [69,70]. EMF has also been shown to regulate the metabolism of specific substances like metallic ions [71]. Interestingly, the nucleoshuttling of ATM was found to be impacted by ionic species like metals [62] or calcium [72]. Hence, while it is too early to hypothesize the action mode(s) of EMF on the nucleoshuttling of ATM, the diversity of physico-chemical reactions that exposure to EMF may produce is consistent with the diversity of phenotypes and symptoms observed in EHS.

**Figure 13:**
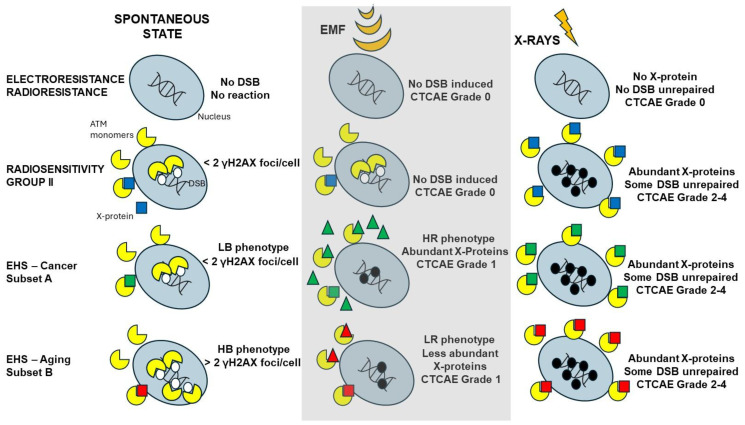
Preliminary model for EHS that is inspired by the RIANS model. For each of the indicated cases of electrosensitivity/radiosensitivity and spontaneously or in response to exposure to EMF or X-rays, schematic illustration of the DSB recognized (white circles) by ATM monomers that lead to repaired (no circle) or unrepaired (black circles). The ATM monomers can enter the nucleus or be sequestrated in cytoplasm by interaction with X-proteins (squares). The X-proteins can be over-expressed spontaneously or by X-ray exposure. The number of unrepaired DSB conditions the degree of severity of the tissue reaction. Such model is based on the hypothesis that EMF does not induce DSB directly but may trigger PTM or changes in the metabolism eventually leading to the release of some metals or calcium ions. All these events may act on ATM (triangles) and delay its nucleoshuttling, which may impact the management of the spontaneous DSB.

At this step, how to explain both LBHR and HBLR phenotypes? In the previous sections, we made the hypothesis that DEMETER subset B (HB phenotype) cells should suffer from a higher yield of endogenous stress than DEMETER subset A (LB phenotype). As already hypothesized above, such features may be caused by heterozygous mutations of certain proteins involved in the response to oxidative stress. With regard to the LR/HR phenotypes, it must be stressed that the final response to any genotoxic stress depends on the amount of repaired DSB and therefore on the DSB recognition. Hence, an HR phenotype can be obtained by a severe lack of DSB recognition caused by EMF-induced PTM or production/liberation of some ions that may be associated with ATM protein and cause a delay in its nucleoshuttling. Conversely, an LR phenotype may require a less severe response to EMF exposure. We endeavored to illustrate in Figure 13 a very preliminary model of EHS in which symptoms are generated from the management of the spontaneous DSB and whose severity is amplified by specific EMF-induced modifications (Figure 13). Obviously, further investigations are required (notably through the exposure of cells to EMF) to validate and document this first model.

## 4. Materials and Methods

### 4.1. The DEMETER Cohort and Fibroblasts Collection

The “Electrosensibles de France/PRIARTEM” association, a non-governmental non-profit French association gathering electrosensitive individuals and promoting research about EHS established a list of self-diagnosed electrosensitive adults (from 18 to 70 years old), members of the association, who can document their discomfort in response to EMF through their family physician. Were notably excluded from the study, pregnant or breastfeeding women, legally protected or deprived of liberty adults. The DEMETER donors agreed to reply to a self-assessment questionnaire and to provide a skin sampling to establish a primary fibroblast cell line. The DEMETER cohort recruitment and the resulting collection of fibroblast cell lines were approved by a regional ethical committee, obeying the French regulations about anonymous sampling and informed consent for medical research. The DEMETER study was promoted by INSERM. The DEMETER donors were sampled between November 2018 and January 2021. A list of medical appointments with a unique dermatologist, clinical officer of the DEMETER study (P.C.), was proposed and donors replied according to their practical availability. The rank order of skin sampling can be therefore considered randomized, it did not influence the questionnaire and the biological data. During each medical appointment, once the information was delivered by the dermatologist and the consent was given by the donor, the donor filled in the questionnaire and sealed it in an envelope.

Thereafter, the dermatologist proceeded to skin sampling in an unirradiated area after local anesthesia with standardized dermatological punch consumables. Briefly, the skin sample was taken by classic dermatological “punch”, on the healthy skin of the inner side of the arm, because less exposed to UV and light. The skin was cleaned with chlorhexidine or alcohol, to the exclusion of any other antiseptic. Local anesthesia by ointment or subcutaneous injection based on lidocaine was currently applied. The biopsy was performed using a dermatome (or “punch”, 2 mm), by cutting the cylindrical fragment of skin with a “23 blade” scalpel using sterile curved Redon forceps, measuring 2 to 3 mm long by 1 mm wide and 1 mm deep. The skin biopsy was immediately placed in a 15 mL tube containing Dulbecco’s modified Eagle’s minimum medium (DMEM) (Gibco-Invitrogen-France, Cergy-Pontoise, France), supplemented with 20% fetal calf serum, penicillin, and streptomycin and sent at room temperature in a double-protected waterproof and shock-resistant transport box in the cell culture lab for cell lines establishing.

Upon receipt, the biopsy was incubated overnight at 4 °C in a 4 mg/mL type II dispase solution (#04942078001; Sigma-Adldrich, Saint-Quentin-Fallavier, France) and the dermis was dissociated from the epidermis using forceps. For fibroblast extraction, the dermis was incubated in a 40 UI/mL collagenase II solution (#C1-28; Sigma-Aldrich) at 37 °C for 90 min and then seeded in the DMEM culture medium described above. The primary cultures thus obtained were amplified in the same culture medium. The routine conditions of the culture of the fibroblasts used in this study were described in Section 4.3.

The self-assessment DEMETER questionnaire was developed by A.M.-P. It was composed of five parts (see Supplementary Table S1 in Supplementary Data):

- (A) questions about the intensities of symptoms and discomfort after exposure to some sources of EMF like cell phone (A1), microwave (A2), cordless landline phone (A3), cell phone base station (A4), household electrical appliance (A5), low-energy light bulbs (A6), wired computer (A7), WiFi-on computer (A8), television (A9), WiFi-on box (A10);
- (B) questions about the intensities of symptoms and discomfort after exposure to some non-EMF sources (see Figure 2C and Supplementary Table S1 for details)
- (C) questions about the intensities of symptoms and discomfort according to the tissue/organ exposed before exposure to EMF sources: eye pathologies (C1), respiratory system (C2), pain in muscles and cartilages (C3), cardiac system pathologies (C4), digestive system pathologies (C5), fatigue and sleep disorders (C6), mood instability and nervousness (C7), slowing down of intellectual activity (C8), headaches, tinnitus (C9), skin pathologies (C10), genito-urinary system pathologies (C11).
- (D) questions about the intensities of symptoms and discomfort according to the tissue/organ exposed during exposure to EMF sources: same as part C.
- (P) a free area for personal information to detail eventual medical history and ongoing treatments.

For A to D questions, intensities were scored from 0 (the lowest intensity) to 5 (the highest intensity).

### 4.2. The COPERNIC Fibroblasts Collection

Since 2004, the UMR1296 Unit staff has collected hundreds of fibroblast cell lines deriving from: (1) group I: apparently healthy individual volunteers, whose radiobiological features clearly indicated radioresistance, low risk of cancer and neurodegenerative disease [40,41] (see also Introduction); (2) group II: cancer patients treated by radiotherapy (RT) and showing a large spectrum of post-RT radiosensitivity tissue individuals reactions; (3) group III: hyper-radiosensitive children suffering from rare and severe genetic diseases. Such a collection, called COPERNIC, is based on both skin sampling and a questionnaire about ongoing treatment and medical history. Since the apparently healthy radioresistant COPERNIC Group I donors did not describe any EHS symptoms in their questionnaire, the COPERNIC Group I fibroblasts and their associated database were considered as radioresistant non-EHS controls for the DEMETER study. Data from 10 COPERNIC Group I fibroblasts were used in this study. Since the COPERNIC Group II fibroblasts with intermediate radiosensitivity did not describe any EHS symptoms in their questionnaire, the COPERNIC Group II fibroblasts and their associated database were considered as radiosensitive non-EHS controls. Data from 186 COPERNIC Group II fibroblasts were used in this study. Lastly, it is noteworthy that radiobiological data from 4 *ATM*-mutated (COPERNIC Group IIIa) and 1 *LIG4*-mutated (COPERNIC Group IIIb) hyper-radiosensitive fibroblast cell lines from non-adult patients were also shown in graphs and tables when necessary. The COPERNIC cohort was composed of 200 cell lines 120 (60%) women and 80 (40%) men. The average age of the donors was 50.4 ± 2.4 years at the skin biopsies sampling (minimum:18; maximum: 79). The legal status of the COPERNIC collection is a bit different from the DEMETER one since the COPERNIC donors were sampled in the frame of their medical care relative to radiotherapy and their cancer treatment. Hence, the COPERNIC collection was approved by a regional ethical committee and also declared under the numbers DC2011-1437 and 2020-3957 to the Ministry of Research as required by the French regulations and was already described elsewhere. It obeys the French regulations regarding anonymous sampling and informed consent [40,41].

### 4.3. Cell Culture

Both DEMETER and COPERNIC cells were kept in nitrogen liquid or cultured in the same location and conditions. Skin fibroblasts were routinely cultured as monolayers in DMEM medium, supplemented with 20% fetal calf serum, penicillin, and streptomycin. Cells were routinely maintained at 37 °C in a humid atmosphere at 5% CO_2_ in flasks for less than 5 days [40,41]. For all the assays described below, confluent cultures were softly detached with 0.025% trypsin and 0.02% ethylenediaminetetraacetic acid (EDTA) (Gibco-Invitrogen-France, Cergy-Pontoise, France) to obtain single cell suspensions that are reseeded for routine culture at dilution 1:2 to 1:4 according to the doubling times. For all the experiments, the cells were seeded 3 days before the experiment in Petri dishes (containing glass slides for immunofluorescence only). All the experiments were performed with cells in the plateau phase of growth (95–99% in G0/G1) to overcome any cell cycle effects. The distribution of cells in the different cell cycle phases was routinely controlled by using flow cytometry [40,41]. With the notable exception of the ATM crowns assay (see below), all the experiments were performed at early passages (i.e., 4 to 10). When needed, the transport of cells to the irradiators was performed in a dedicated safety-cooled box.

### 4.4. X-Rays Irradiation

All the irradiations were performed on a 6 MeV X-ray clinical irradiator (SL 15 Phillips) at the anti-cancer Centre Léon-Bérard (Lyon, France) at a dose of 2 Gy with a dose rate of 6 Gy.min^−1^. Dosimetry was certified by the Radiophysics Department of Centre Léon-Bérard [40,41].

### 4.5. Treatment with H_2_O_2_

Hydrogen peroxide (H_2_O_2_; manufacturer) treatment consisted of adding the indicated concentration of H_2_O_2_ directly to the culture medium for 30 min. Thereafter, the culture medium was removed and cells were rinsed with physiological serum (0.9% NaCl) [47].

### 4.6. Pre-Treatment with Anethole Trithione

Anetholetrithione (AOL) (#SML4108; Sigma-Aldrich (Saint-Quentin-Fallavier, France)) is known to be an efficient antioxidative agent [48,49,50]. AOL was added directly into the culture medium for 24 h to reach a final concentration of 10 µM AOL. Thereafter, the medium was removed and cells were rinsed with physiological serum (0.9% NaCl).

### 4.7. Immunofluorescence

Immunofluorescence and foci scoring procedures were described elsewhere [40,41,46]. Briefly, anti-*γH2AX^ser139^* antibody (clone JBW301; Merck, Millipore, Darmstadt, Germany) was applied at 1:800. Anti-*pATM^ser1981^* (clone 10H11.E12; Millipore, Darmstadt, Germany), and anti-*MRE11* (#56211; Abcys, Paris, France) were used at 1:100. Cells were counterstained with 4′,6-Diamidino-2-Phenylindole, Dihydrochloride (DAPI), which also permit to score the micronuclei in the same conditions (see below). The Foci scoring procedure applied here has received the certification agreement of CE mark and ISO-13485 quality management norms and developed some features protected in the frame of the patents FR3017625 A1 and FR3045071 A1 [40,41].

### 4.8. Micronuclei Assay

During each immunofluorescence experiment, the DAPI counterstaining permits the quantification of the micronuclei at magnification X100 [40,41].

### 4.9. Statistical Analysis

With regard to the self-assessment questionnaire, data analysis was based on the calculation of simple averages of the grading of symptoms or discomfort intensity for a given wave frequency (Part A), a given source of stress (part B), or a given tissue/pathology (Parts C and D). Furthermore, the definition of matching ratios (defined in the text) facilitated the identification of the common features of subsets. We have developed an analytic approach for each questionnaire parts A to D. Such a step-by-step approach permits us to avoid any artifacts by favoring crossed comparisons with more than one criterion, at least. A color code applied in the tables facilitated the synthesis of the findings.

With regard to the biological data analysis, the ANOVA test was used to compare two means, and Spearman’s test was used to compare the kinetic data. All data were obtained with the numbers of independent experiments indicated and each value is expressed as its mean and standard error to the mean (Poisson’s law).

In all the studies, the systematical determination of mathematical functions linking to endpoints was privileged to facilitate the development of the kinetic and mechanistic models. The correlation coefficient providing the quality of fit was systematically provided. The statistical analyses were performed either by PRISM software version 8 (GraphPad Software, San Diego, CA, USA) or by using Kaleidagraph v4 (Synergy Software, Reading, PA, USA).

## 5. Conclusions

EHS and its causal link with exposure to EMF remain a major question of public health. By considering that behavior studies or those based only on a self-assessment questionnaire may be a source of numerous biases, we designed a clinical study gathering 26 adult volunteers self-diagnosed as electrosensitive (EHS) to propose a molecular definition of EHS (DEMETER) by combining a self-assessment questionnaire and biological investigations on a collection of primary fibroblasts derived from the DEMETER patients. Both questionnaire and biological data were analyzed independently. The questionnaire and the biological data revealed 2 subsets of donors characterized each by a specific phenotype: a low background and high responsiveness (LBHR) or else a high background and low responsiveness (HBLR) phenotype. The highest self-assessed intensities of discomfort were obtained for cerebral features like headaches, tinnitus, slowing down of intellectual activity, fatigue, and sleep disorders. The HBLR phenotype was found characterized by very high amounts of spontaneous DSB and MN, like those of the most hyper-radiosensitive cell lines while the LBHR phenotype was found associated with limited amounts of spontaneous DSB and MN, like cells from patients showing moderate radiosensitivity. By applying X-rays, all the DEMETER cell lines (26/26) appeared characterized by delayed radiation-induced ATM nucleoshuttling (RIANS). However, it must be stressed that a given patient showing delayed RIANS does not necessarily suffer from EHS. The LBHR phenotype was associated with an early formation of radiation-induced MRE11 foci and high cancer risk while the HBLR phenotype was associated with both spontaneous formation of perinuclear pATM crowns and late formation of radiation-induced MRE11, common features of high risk of accelerated aging. Treatment with H_2_O_2_, a DNA strand-break inducer, led to the formation of HDC at a higher rate for the HBLR than for the LBHR phenotypes. Interestingly, exposing DEMETER cells to H_2_O_2_ treatment, followed by an antioxidative treatment resulted in a decrease in the number of HDCs. Such findings confirmed that EHS may be related to the management of SSB and/or DSB. A very preliminary model of EHS inspired by the RIANS model was proposed. These conclusions are based on in vitro cellular experiments and require further studies to verify the applicability of these findings to EHS individuals. Furthermore, directions for future studies, such as in vivo experiments or larger cohort studies, can be proposed to further validate the findings.

## Figures and Tables

**Table 1:** Quantitative features of the questionnaire parts C and D according to the organ/pathology.

	Average C Intensity	Average D Intensity	D/C Ratio	NR * (%)	LBHR * Matching (%)	HBLR * Matching (%)
Eyes	1.16	2.62	2.25	48	40	12
Respiratory system	1.11	2.08	1.87	48	44	8
Muscles and cartilages	1.48	3.44	2.32	48	44	8
Cardiac system	1.00	3.32	3.32	36	48	16
Digestive system	1.46	2.88	1.97	32	60	8
Fatigue, sleep disorders	2.00	4.16	2.08	32	32	36
Mood instability nervousness	1.26	3.20	2.54	28	40	32
Intellectual activity	1.50	4.04	2.69	24	36	40
Headaches, tinnitus	1.23	4.40	3.57	28	48	24
Skin	0.77	2.84	3.68	40	56	4
Genito-urinary system	0.88	2.00	2.27	52	40	8

* The cells were stained in red, green, or grey if HBLR, LBHR phenotypes, or null responses represent the majority of cases for a given organ/pathology, respectively.

**Table 2:** Quantitative features of the questionnaire parts C and D according to the organ/pathology and the DEMETER donors.

Organ/ Donor	Eyes	Respiratory Sys.	Muscles	Cardiac Sys.	Digestive Sys.	Sleep Disorder	Mood Instability	Intellect. Activity	Headaches, Tinnitus	Skin	Genital Sys.	NR * (%)	LBHR *(%)	HBLR *(%)	Subset 1′/2′	Subset 1/2
1												18.1	45.4	36.3	45.4	
2												81.8	9.1	9.1	81.8	
3												9.1	0	81.8	81.8	
4												63.6	36.3	0	63.6	
5												63.6	18.1	18.1	63.6	
6												45.4	36.3	18.1	45.4	
7												0	100	0	100	
8												72.7	27.2	0	72.7	
9												36.3	36.3	27.2	36.3	
10												18.1	45.4	36.3	45.4	
11												72.7	9.1	18.1	72.7	
12												0	9.1	91	91	
13												27.2	72.7	0	72.7	
14												0	72.7	27.2	72.7	
15												54.5	45.4	0	54.5	
16												27.2	72.7	0	72.7	
17												27.2	81.8	0	81.8	
18												36.3	0	63.6	63.6	
19												9	91	0	91	
20												72.7	9.1	18.1	72.7	
21												27.2	63.6	9.1	63.6	
22												63.6	27.2	9.1	63.6	
23												91	0	9.1	91	
24																
25												18.1	36.3	45.4	45.4	
26												18.1	36.3	45.4	45.4	

* The cells were stained in red, green, or grey if HBLR, LBHR phenotypes, or NR represent the majority of cases for each organ/pathology, respectively.

**Table 3:** γH2AX foci data from DSB repair kinetics.

	2 Gy + 10 min	2 Gy + 24 h
COPERNIC Group I (controls) [40]	79.0 ± 4.0	0 ± 1
DEMETER	38.4 ± 1.1	1.68 ± 0.14
DEMETER Subset 1 (LBHR)	37.8 ± 2.2	1.52 ± 0.24
DEMETER Subset 2 (HBLR)	38.7 ± 1.1	1.80 ± 0.17
DEMETER Subset A (LBHR)	39.5 ± 1.6	1.36 ± 0.14
DEMETER Subset B (HBLR)	36.4 ± 0.3	2.19 ± 0.23
COPERNIC Group II [40]	15–60	0–8
COPERNIC Group IIIa [40]	6.4 ± 3.2	0 ± 1
COPERNIC Group IIIb [40]	78.0 ± 2.0	36.6 ± 4.2

**Table 4:** pATM foci data from DSB repair kinetics.

	2 Gy + 10 min	2 Gy + 24 h
COPERNIC Group I (controls) [40]	40.2 ± 2.2	0 ± 1
DEMETER	23.9 ± 1.0	2.30 ± 0.18
DEMETER Subset 1 (LBHR)	21.9 ± 1.6	2.02 ± 0.22
DEMETER Subset 2 (HBLR)	24.9 ± 1.4	2.05 ± 0.27
DEMETER Subset A (LBHR)	23.5 ± 1.4	2.00 ± 0.40
DEMETER Subset B (HBLR)	24.7 ± 1.2	2.86 ± 0.45
COPERNIC Group II [40]	8–30	0–4
COPERNIC Group IIIa [40]	3.2 ± 2.8	0 ± 1
COPERNIC Group IIIb [40]	39.2 ± 3.0	17.4 ± 4.2

**Table 5:** MRE11 foci data from DSB repair kinetics.

	2 Gy + 1 h/4 h	2 Gy + 24 h
COPERNIC Group I (controls) [45]	5–7	0 ± 0
DEMETER	5.52 ±.0.56/4.0 ± 0.45	0.47 ± 0.19
DEMETER Subset 1 (LBHR)	5.88 ± 0.67/3.22 ± 0.76	0.18 ± 0.18
DEMETER Subset 2 (HBLR)	5.25 ± 0.87/4.58 ± 0.51	0.66 ± 0.28
DEMETER Subset A (LBHR)	6.2 ± 0.57/3.8 ± 0.51	0 ± 0
DEMETER Subset B (HBLR)	3.66 ± 1.1/4.5 ± 1.11	1.66 ± 0.33
COPERNIC Group II [45]	5–12/5–0	0–5
COPERNIC Group IIIa [45]	Impaired MRE11 foci	Impaired MRE11 foci
COPERNIC Group IIIb [45]	Impaired MRE11 foci	Impaired MRE11 foci

**Table 6:** General features of the data fits shown in Figure 10.

	Parameter a *	Parameter b * in μM	Correlation Coefficient r
COPERNIC Group I (controls)	0	0	1
COPERNIC Group II	22.8 ± 1.7	139 ±38	0.995
DEMETER 22 (subset A)	29.1 ± 2.8	27 ± 13	0.984
DEMETER 14 (subset B)	58.0 ± 8.1	167 ± 83	0.982
DEMETER 15 (subset B)	66.1 ± 4.8	110 ± 29	0.994
DEMETER 17 (subset B)	99.3 ± 6.0	240 ± 48	0.998
DEMETER 23 (subset B)	80.8 ± 39.6	618 ± 125	0.973
DEMETER 25 (subset B)	113.0 ± 26.3	144 ± 69	0.945

* from the fitting formula: y = ax/(b + x).

**Table 7:** Summary of the composition of the subsets defined in this study and the corresponding matching.

Donor	Sex	Subsets 1/2 from Part A	Subset 1′/2′ from Parts C and D	Subset A/B from Spontaneous Data	Subset A/B from MRE11 Data	Matching * Subsets 1/2 vs. 1′/2′	Matching ** Spontaneous vs. MRE11 Data	Matching *** Subsets1/2 vs. A/B
01	M					0%	100%	50%
02	M					50%	100%	50%
03	M					100%	100%	50%
04	F					50%	50%	50%
05	F					50%	100%	75%
06	F					50%	50%	0%
07	F					0%	100%	75%
08	M					50%	100%	75%
09	M					100%	100%	100%
10	F					0%	100%	75%
11	F					50%	100%	75%
12	F					100%	100%	50%
13	F					0%	100%	50%
14	F					0%	100%	100%
15	F					50%	50%	0%
16	F					100%	100%	75%
17	F					0%	50%	75%
18	F					0%	100%	75%
19	F					0%	100%	75%
20	F					50%	100%	75%
21	F					0%	100%	100%
22	F					50%	100%	75%
23	M					50%	100%	50%
24	F					nc	100%	nc
25	F					0%	100%	50%
26	F					100%	100%	75%
Mean						40%	92.3%	64%

* A score of 100% and 50% was attributed for 1 color + grey, and for green + red, respectively; nc: non-considered; ** A score of 100%, 50% or 0% was attributed for 2 same colors, for 1 color + orange, for green + red, respectively; *** A score of 100%, 75%, 50% or 0% was attributed for 4 same colors, for 3 same colors, +grey, for green + red, respectively; Grey, black and orange cases indicate null response (NR), no calculation possible (donor#24), and “zero-function” shape for the MRE11 foci kinetics.

**Table 8:** Major features of the LBHR/HBLR phenotypes from the questionnaire and biological data analysis.

Data Source	Endpoint	LBHR Phenotype	HBLR Phenotype
Questionnaire data	Part A	Subset 1: LB if I_A5,A6,A7_ < 2.48 HR if I_average_ (UHF) − I_A5,A6,A7_ = 2.7	Subset 2: HB if I_A5,A6,A7_ ≥ 2.48 LR if I_average_ (UHF) − I_A5,A6,A7_ = 0.15
	Part B	No particular sensitivity to other chemical or physical agents than EMF sources	Sensitivity to other chemical or physical agents than EMF sources Link to immunodeficiency?
	Part P	28.6% LBHR donors mentioned ongoing treatment and/or medical history	71.4% HBLR donors mentioned ongoing treatment and/or medical history
	Part C,D	Subset 1′: matched with subset 1 at 40% impairment of cardiac and digestive systems, mood instability, nervousness, headaches, tinnitus and skin reactions	Subset 2′: matched with subset 2 at 40% fatigue, sleep disorders and the decrease of the intellectual capacity
Biological data	Spontaneous γH2AX and MN	Subset A: <2 γH2AX foci per cell Subset B: <5 MN per 100 cells matched with subset 2 at 64%	Subset A’: ≥2 γH2AX foci per cell Subset B’: ≥5 MN per 100 cells matched with subset 2 at 64%
	Perinuclear pATM crowns	No perinuclear pATM crowns at high culture passages	Perinuclear pATM crowns at high culture passages
	X-rays-induced γH2AX foci kinetics	Delayed RIANS	Delayed RIANS
	X-rays-induced pATM foci kinetics	Delayed RIANS	Delayed RIANS
	X-rays-induced MRE11 foci kinetics	Early MRE11 foci (high risk of cancer)	Late MRE11 foci (high risk of accelerated aging)
	H_2_O_2_-induced γH2AX foci	Low yield of HDC	High yield of HDC

## Data Availability

All the data can be provided on reasonable request.

## Supplementary Materials

The following supporting information can be downloaded at: https://www.mdpi.com/article/10.3390/ijms26104792/s1.
