# Supplementary material for: Skin Fibroblasts from Individuals Self-Diagnosed as Electrosensitive Reveal Two Distinct Subsets with Delayed Nucleoshuttling of the ATM Protein in Common
Source: Int J Mol Sci. 2025 May 16;26(10):4792. doi: 10.3390/ijms26104792 (PMC12112057; doi:10.3390/ijms26104792)
Supplement: Supplementary file 1 [file ijms-26-04792-s001.zip › ijms-3595102-supplementary.pdf]

Skin fibroblasts from individuals self-diagnosed as electrosensitive reveal two distinct subsets with delayed nucleoshuttling of the ATM protein in common

SUPPLEMENTARY DATA

Table S1: Summary of the part P of the questionnaire (ongoing treatment and medical history)

Donor	Sex	Subset	Ongoing treatment	Medical history
01	M	2		
02	M	2		
03	M	2		
04	F	1		
05	F	1		
06	F	2	Beta-blocker, antiasthmatic, antihistamine	High blood pressure
07	F	1		
08	M	1		
09	M	1		
10	F	2		
11	F	1		
12	F	2		Breast cancer
13	F	2		
14	F	2		
15	F	1		Zona
16	F	1		
17	F	2	Beta-blocker, blood thinner	Breast cancer<br>Anaphylactic shock
18	F	1		
19	F	2	Iron compound medecine, treatment against hypothyroidism and against cognitive trouble	Iron Anemia,<br>Hypothyroidism<br>Memory trouble
20	F	1		
21	F	2		
22	F	1		
23	M	1		
24	F	1		
25	F	1	anxiolytic	
26	F	2	Beta-blocker, anxiolytic, antihypertensive	Thalidomide poisoning and hemiplegia (during chilhood)<br>High blood pressure<br>Arthrosis

*Defined from the value of $I_{A5,A6,A7}$ (see section 3.1.2)

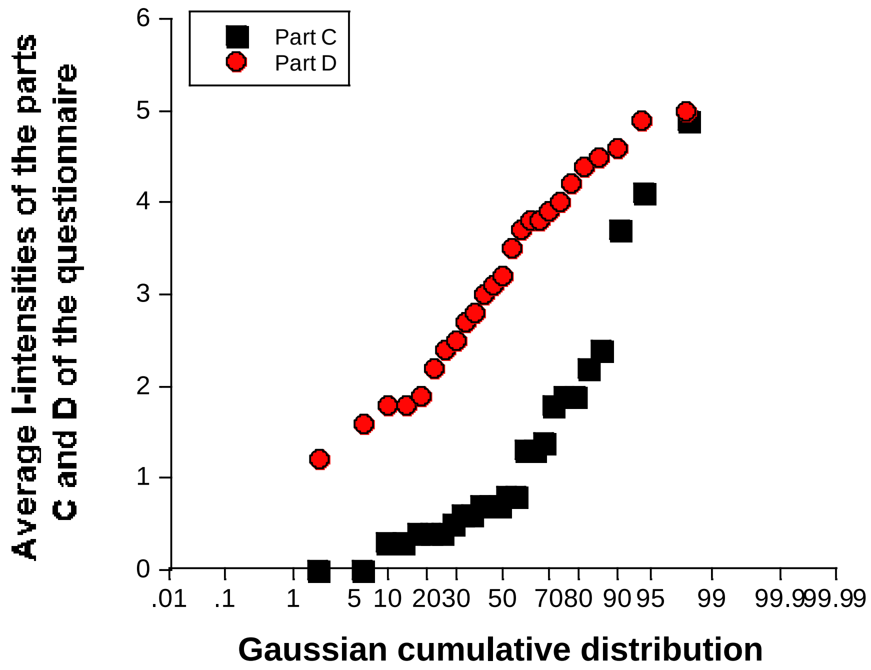

Figure S1: Gaussian cumulative distribution of the average I-intensities of the parts C and D of the self-assessment questionnaire. The sigmoidal shape of the of each series of data indicate that the average I-intensities obey the Gaussian probability function.

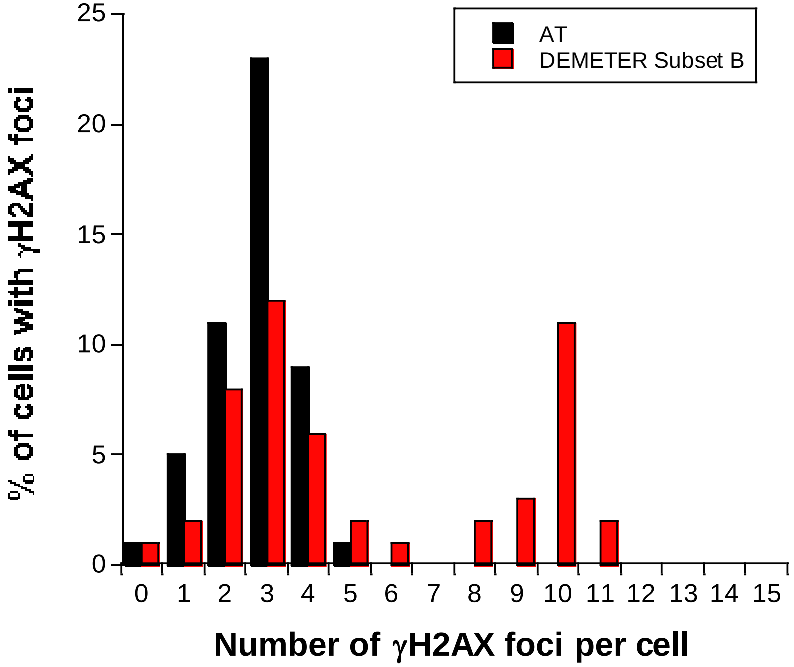

Figure S2: Spontaneous γH2AX foci distribution in representative ATM-mutated and DEMETER subset B fibroblasts. Each value is the pool of at least 2 independent replicates.

DEMETER
SELF-ASSESSMENT QUESTIONNAIRE

PART A: Grade (0 - 5) the intensity of your symptoms or discomfort according to the sources of risk

1. Using a cell phone
2. Using a microwave
3. Using a cordless landline phone
4. Presence of a cell phone base station
5. Using a household electrical appliance
6. Using of low-energy light bulbs
7. Using a wired computer
8. Using a wifi computer
9. Using a television
10. Using a wifi box

PART B: Grade (0 - 5) the intensity of your symptoms or discomfort according to the following sources other than EMF.

1. Some food (milk, bread, meat, spiced food or food additives like glutamate)
2. Caffeine (coffee, tea, sod, others caffeinated products) or chocolate
3. Noises or sounds usually tolerated by others
4. Some medicines
5. Other medical, surgical or dental equipment or procedures
6. Household cleaning products (detergents, coating, exhaust gas…)
7. Perfumes, cosmetics, home fragrances
8. Allergies to pollen, dust, mould, insect bites and food
9. Notion of discomfort after eating
10. Wearing certain types of clothing, skin contact with certain fabrics

PART C : Grade (0 - 5) the intensity of your symptoms or discomfort according to the following organs/pathologies before exposure to EMF

1. Visual problems: dryness, tearing, burning or irritation of the eyes
2. Respiratory problems: breathlessness, coughing, phlegm, respiratory infections
3. Muscle or joint pain, cramps, stiffness or weakness
4. Heart or chest problems: palpitations, rapid heartbeat, heart rhythm problems or chest discomfort
5. Digestive problems: abdominal pain, cramps, bloating, nausea, transit problems: diarrhea or constipation
6. Fatigue, sleep disorders
7. Mood instability, tension or nervousness, irritability, depression, crying spells or outbursts of anger, lack of interest in activities that are usually motivating
8. Slower intellectual activity: difficulty concentrating, memory problems, difficulty making decisions
9. Headache or feeling of heavy head or congested face, tinnitus
10. Skin problems: burning, tingling, rashes, redness
11. Genitourinary problems: pelvic pain or the need to urinate frequently or urgently (for women: discomfort or other problems related to menstruation)

PART D: Grade (0 - 5) the intensity of your symptoms or discomfort according to the following organs/pathologies during exposure to EMF

1. Visual problems: dryness, tearing, burning or irritation of the eyes
2. Respiratory problems: breathlessness, coughing, phlegm, respiratory infections
3. Muscle or joint pain, cramps, stiffness or weakness
4. Heart or chest problems: palpitations, rapid heartbeat, heart rhythm problems or chest discomfort
5. Digestive problems: abdominal pain, cramps, bloating, nausea, transit problems: diarrhea or constipation
6. Fatigue, sleep disorders
7. Mood instability, tension or nervousness, irritability, depression, crying spells or outbursts of anger, lack of interest in activities that are usually motivating
8. Slower intellectual activity: difficulty concentrating, memory problems, difficulty making decisions
9. Headache or feeling of heavy head or congested face, tinnitus
10. Skin problems: burning, tingling, rashes, redness

11. Genito-urinary problems: pelvic pain or the need to urinate frequently or urgently (for women: discomfort or other problems related to menstruation)

PART P : Detail your ongoing treatment and medical history
